# Prediction of Elution Profiles in Countercurrent Chromatography With a Linear Loss of Stationary Phase

**DOI:** 10.1002/jssc.70526

**Published:** 2026-09-13

**Authors:** Felix Rüttler, Rosalie Ormos, Walter Vetter

**Affiliations:** ^1^ Department of Food Chemistry (170b) Institute of Food Chemistry University of Hohenheim Stuttgart Germany

**Keywords:** bleed, carotenoid, countercurrent chromatography, ProMISE, *S_f_* value

## Abstract

Countercurrent chromatography (CCC) is a liquid‐liquid separation method that is predominantly used for the preparative isolation of natural products. Key indicators for the development of CCC methods are the partition coefficient (*K* value) between the two liquid phases and the properties of the chosen biphasic solvent system. Based on this information, it is possible to predict elution volumes and peak widths with the probabilistic model for immiscible separations and extractions (ProMISE). However, a common challenge in reliably predicting CCC elution profiles is a persistent loss of stationary phase (i.e., bleeding), which leads to a decrease in the percentile share of stationary phase (*S_f_* value) in the column as the separation progresses. In this study, two carotenoids were isolated and used as test compounds to investigate the effect of bleeding with the *n*‐hexane/benzotrifluoride/acetonitrile (20:7:13, *v*/*v*/*v*) solvent system. After showing that the stationary phase bleeding rate was linear (and not exponential), experiments were carried out both in head‐to‐tail and in tail‐to‐head mode. Next, a simple calibration method was developed to predict elution volumes and peak widths with the ProMISE2 software in CCC separations with linear bleeding. Finally, based on the collected data, a method was developed to accurately predict the elution volume and peak width during a (long) CCC run, particularly for analytes with comparatively high elution volumes.

## Graphical abstract

A common challenge in reliably predicting elution profiles in countercurrent chromatography using the ProMISE2 software is a persistent loss of stationary phase (i.e., bleeding). This drawback can be overcome by a simple calibration method in the case of linear bleeding.

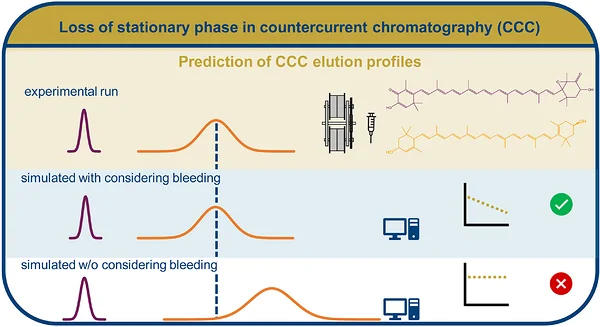

**Abbreviations**
^1^H NMR: proton magnetic resonance spectroscopy
BTF: benzotrifluoride
BTF system: *n‐*hexane/benzotrifluoride/acetonitrile (20:7:13, *v*/*v*/*v*)
CCC: countercurrent chromatography
DCM: dichloromethane
LC‐MS: liquid chromatography with mass spectrometry
ProMISE: probabilistic model for immiscible separations and extractions.

## 1 Introduction

Invented by Ito et al. in 1966 [1], countercurrent chromatography (CCC) is a comprehensive term for all liquid‐based centrifugal separations without solid support [2]. The term “countercurrent” is somewhat misleading, as it describes a theoretical phenomenon responsible for the method's functionality [3, 4]. In practice, a biphasic solvent system is used with one liquid phase being held stationary by centrifugal forces in a multilayer coil, and the other, mobile phase, is continuously pumped through it [5]. Analytes are separated in the coil through mass transfer [6, 7], i.e., transport with the mobile phase and countless mixing/demixing steps between the mobile and stationary phase according to the partition coefficient (*K* value) [8]. The importance of CCC in the isolation of polar natural products is steadily increasing, given the wide range of hydrophilic biphasic solvent systems available [9]. However, there are few suitably hydrophobic solvent systems, particularly for lipid compounds, highlighting the relevance of their reliable use [10].

Although many instrumental (coil dimension [11, 12], rotor speed [13, 14]) and experimental factors (the selectivity of the biphasic solvent system [5, 15], the retention of the stationary phase (*S_f_* value) [13, 16], mixing and demixing [6, 17], flow rate [13, 18], and temperature [5, 12]) contribute to the quality of CCC separations, the overall performance can be predicted with the “probabilistic model for immiscible separations and extractions” (ProMISE) [19, 20, 21]. ProMISE is an iterative model for describing CCC separations, which models device‐specific peak widths as a function of the so‐called efficiency factor *f'* [19, 21]. Factor *f'* is a dimensionless value for including mixing/settling efficiency and device‐specific parameters of a countercurrent chromatographic device [19]. Therefore, factor *f'* should not be confused with the plate number *N* used to describe efficiency in the chromatographic plate model [19]. The peak width at base *w_b_* can be written as the standard deviation 4*σ* [min] of probabilistic units as a function of the elution time *t* [min], the *K* value [‐], the phase distribution *X* [‐], the rotor speed *ω* [1/min], and factor *f'* [‐], with 4*σ* = 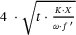 [19].

However, several effects can impair the predictability of CCC separations, with persistent loss of (minute amounts of) stationary phase (i.e., bleeding) [22] being the most common and most relevant case. Since the *S_f_* value is a fixed input variable (a constant) in the ProMISE model, loss of stationary phase inherently leads to wrong predictions of elution times and peak widths [21]. Accordingly, a stable biphasic solvent system (no bleeding) is a prerequisite for the direct application of the ProMISE model [21]. In line with practical experience gained during the development of the hydrophobic BTF system, i.e., *n*‐hexane/benzotrifluoride/acetonitrile (20:7:13, *v*/*v*/*v*) [10, 23, 24, 25, 26, 27, 28], a recent study has revealed unexpected shifts in *K* values in comparatively long CCC separations, which were likely caused by bleeding [29]. Once a small share of the stationary phase is lost after the initial equilibration of the solvent system, this effect increases proportionately to the elution time [21, 22, 30].

The present study aimed to investigate the phenomenon of bleeding exemplarily with the BTF system and to overcome it by including it in theoretical considerations to improve the predictability of the ProMISE model in such a situation. Due to their good detectability and challenging *K* values [23], the carotenoids lutein and astaxanthin were initially selected as analytes because they could be isolated in sufficient amounts from active ingredients (safflower oils). Following CCC fractionation and structural analysis, it became apparent that the main compounds in the safflower oils examined were lutein and (surprisingly) astaxanthin‐5,6‐epoxide. Together with lutein, astaxanthin‐5,6‐epoxide was found to be a very interesting test compound for this study. Next, a simple calibration was developed that considered the gradual loss of the stationary phase and enabled correction of the *S_f_* value and the *K* values during CCC separations. This approach was tested with different scenarios with the present CCC device.

## 2 Materials and Methods

### 2.1 Samples, Solvents, and Chemicals

Commercially available samples enriched in lutein (20%, liquid in safflower oil, hereafter lutein oil) and astaxanthin (5%, *Haematococcus pluvialis* extract in safflower oil, hereafter astaxanthin oil) were ordered online (July 2025). Astaxanthin (crystalline) was provided by Alexis (Lausen, Switzerland). The sources of the solvents and chemicals used were previously reported (Section S1) [27, 31].

### 2.2 Spectrophotometric Determination of Partition Coefficients (*K* Values)

*K* values of the lutein oil (20% lutein according to label) and the astaxanthin oil (5% astaxanthin according to label) were determined spectrophotometrically according to Ito et al. [5], with slight modifications (Section S2a). Solvent influence on the absorption maximum (*λ*_max_) and band shapes was checked in advance, with *λ*_max_ = 444 nm being determined for the lutein oil and *λ*_max_ = 474 nm for the astaxanthin oil (Figure S1). *K* values were calculated from the concentrations of the analytes in the stationary and mobile phases (*c*_*s*/*m*_), i.e., the ratio of *λ*_max_ either between the upper and lower phases (here: *K*_*U*/*L*_ value) or lower and upper phases (here: *K*_*L*/*U*_ value), based on Equation (1) [5]:

(1) $$K=c_{s\;}/\;c_{m}$$

Later, the method for determining *K* values was repeated with astaxanthin oil, with three further variations of the BTF system being investigated (Section S2b).

### 2.3 Countercurrent Chromatography

CCC separations were conducted with a QuikPrep MK8 device (AECS, St Columb Major, Cornwall, UK) (Figure S2) according to Rüttler and Vetter (Section S3a) [21]. All CCC separations were performed with the solvent system *n*‐hexane/BTF/ACN (20:7:13, *v*/*v*/*v*) with different coils either in head‐to‐tail (lower phase mobile) or tail‐to‐head mode (upper phase mobile) by selecting different settings on the device's multi‐channels (Figure S3). First, the selected coil(s) were filled with the stationary phase, followed by equilibration with mobile phase for 20 min. The displaced stationary phase was collected with a graduated cylinder, and the volume was exactly measured using a burette (Duran, Wertheim, Germany, ± 0.075 mL Ex 20°C). In this study, the *S_f_* value was treated as a time‐dependent variable and is henceforth defined as *S_f_* (*t*) (in %, where *t* denotes time in min). *S_f_* (*t*) was calculated from the measured volume of stationary phase in the coil, which was also time‐dependent and defined as *V_s_* (*t*) (in mL, where *t* denotes time in min), and the coil volume (*V_c_*), which was constant (Equation 2) [13]:

(2) $$S_{f}\;(t)=(V_{s}\;(t)\;\cdot \;100)/(V_{c})$$

Subsequently, *S_f_* (0) was calculated, and the samples were injected and dissolved in 4.5 mL upper and lower phases. The UV/vis signal of the effluent was monitored at *λ* = 400, 444, 474, and 520 nm, and the sample was fractionated as described below (Sections 2.3.1, 2.3.3, and 2.3.4). After each CCC run (exception: enrichment of test compounds, Section 2.3.1), the respective coils were flushed with nitrogen, the effluent was collected in a graduated cylinder, *V_s_* (70, 300, or 320) was determined with a burette, and *S_f_* (70, 300, or 320) was calculated (Equation 2).

#### 2.3.1 CCC Enrichment of the Test Compounds

Coils 2+3 (*V_c_* = 236 mL, Figure S3f) were used for separations in head‐to‐tail mode (lower phase mobile), with both phases being pumped synchronously with the same flow rate (mobile phase, $\dot{v}$_*m*_ = 2 mL/min and stationary phase, $\dot{v}$_*s*_ = 2 mL/min, respectively) to the coil outlet (co‐current mode) [32, 33], to enrich lutein and astaxanthin‐5,6‐epoxide from the respective solution in safflower oil. Due to the much higher share of stationary phase in the coils (*S*_*f*_ (0) ≈ 80%), the mobile phase was moved considerably faster than the stationary phase [34, 35]. Under these conditions, the stationary phase was fully replaced after ∼90 min.

In practice, the biphasic solvent system was equilibrated for 20 min with mobile phase (determination of *S*_*f*_ (0)), and both phases were then pumped for 10 min in co‐current mode. Afterward, ∼260 mg sample was injected every 70 min, with the lutein oil being separated once and the astaxanthin oil three times (4 × separations in sequence). The effluent was monitored as described above (Section 2.3), and the respective fractions (lutein oil: fraction L1, 20–30 min; astaxanthin oil (detector signal indicated the presence of several compounds): fractions A1 + A2, 148–153 + 153–165 min, 218–223 + 223–235 min, and 288–294 + 294–305 min) were collected in amber round‐bottom flasks. All collected fractions with analytes were rotary evaporated (80 mbar/40°C) to 1 mL and transferred with dichloromethane (DCM) into pre‐weighed amber 20 mL screw‐top glasses. The solvent was removed under a gentle nitrogen stream, the fraction yield was determined, and the dry residue was taken up in 10 mL of DCM. These stock solutions were stored at 8°C in the dark for further experiments.

#### 2.3.2 Modelling the Gradual Loss of Stationary Phase

##### 2.3.2.1 CCC Separations

Experiments to model the loss of stationary phase [22] during the use of the BTF system were carried out with coil 1 (*V_c_* = 116 mL) in head‐to‐tail (Figure S3a) and tail‐to‐head mode (Figure S3e) with $\dot{v}$ *_m_* = 2 mL/min, respectively. First, coil 1 was filled with the respective stationary phase and equilibrated with mobile phase for 20 min (determination of *S_f_* (0)). Thereafter (without sample injection), sixteen 20 min fractions (elution time 320 min) were taken, and the volumes of the mobile and the displaced stationary phase (*V*_displaced_) were determined with a burette (Duran) for each fraction [36]. At the end of CCC separations, the coil was flushed with nitrogen, and *S_f_* (320) was determined.

##### 2.3.2.2 Data Evaluation

The data obtained were transferred to Excel and treated as follows: (i) *S_f_* (*t*) was calculated according to Equation (2) using the experimental values for the decrease in the stationary phase (*V_s_* (*t*) = *V_s_* (0) ‐ *V*_displaced_), (ii) plotted against the elution time (*t*), and (iii) linearly regressed. Each set of 18 data points formed the graph for *S_f_* (*t*) in the range between 0 and 320 min. (iv) A linear regression analysis was performed using Excel's LINEST function to determine the slope (in %/min), the standard error of the slope, and the coefficient of determination (*R* ^2^).

#### 2.3.3 Determination of Efficiency Factor *f*'

##### 2.3.3.1 CCC Separations

The efficiency factor *f'* for different coil combinations and elution modes (Figure S3) was determined at $\dot{v}$ = 2 mL/min and with an analyte dosage of 0.5 mg lutein and 2.0 mg of astaxanthin‐5,6‐epoxide (from stock solutions, Section 2.3.1) dissolved in 4.5 mL of the upper and lower phases. The respective method elution time was determined prior to CCC separations based on the determined *K* values of the analytes and simulation using ProMISE2 software (Figure S4) [21]. In the next step, *S_f_* (0) was determined, and runs with both test compounds were carried out for 70 min in head‐to‐tail mode (number of experiments: four separate triple determinations, *n* = 12). However, in tail‐to‐head mode, the elution time was 300 min for one coil (number of experiments: two separate triple determinations, *n* = 6). In both modes, the entire effluent was collected in an amber round‐bottom flask; the solvent was removed using rotary evaporation (80 mbar/40°C), and the test compounds were redissolved in 4.5 mL of the upper and lower phases and reused for the next CCC separation. After each CCC separation, the coil was flushed with nitrogen as described above (Section 2.3.2), and *S_f_* (70) or *S_f_* (300) was determined.

##### 2.3.3.2 Data Evaluation

The elution times (*t*) and peak widths at base (*w_b_*) of both test compounds were directly taken from the CCC‐UV/vis chromatograms at 474 nm [21] and entered into Excel along with the calculated values for *S_f_* (start) and *S_f_* (end). A linear regression of *S_f_* (*t*) (Section 2.3.2), comprising two data points for start and end, was plotted as a function of the elution time *t* (Equation 3). Based on the slope of the decrease in the percentile share of stationary phase *s* and the intercept *S_f_* (start), *S_f_* (*t*) could now be calculated for an arbitrary elution time *t* (Equation 3):

(3) $$\begin{array}{ccc} S_{f}\left(t\right) & = & ((S_{f}\;(\mathrm{end})\;-\;S_{f}\;(\mathrm{start}))/(\mathrm{end}))\;\cdot t+S_{f}\;(\mathrm{start})\\ & = & s\cdot t+S_{f}\;(0)\; \end{array}$$

where the start was defined as *t* = 0 min and the end as *t* = 70 or 300 min to obtain the experimental values for *S_f_* (start) = *S_f_* (0) and *S_f_* (end) = *S_f_* (70) or *S_f_* (300).

Based on the experimental runs, the elution times *t* of the test compounds in head‐to‐tail or tail‐to‐head mode were known (see above) and could be substituted into the function of *S_f_* (*t*) (Equation 2) to calculate *S_f_* (*t*) at the maxima of the test compounds. Using the calculated value of *S_f_* (*t*) for both test compounds and the elution volume ($V_{r}=t\cdot {\dot{v}}_{m}$), the corresponding *K* value at the maximum could be derived (Equation 4) [5]:

(4) $$K=\frac{(t\cdot {\dot{v}}_{m})\;-\;V_{c}\cdot \;((100\;-\;S_{f}\;(t))/\left(100)\right)}{V_{c}\cdot \;((S_{f}\;(t))/(100))}$$

The calculated values for *S_f_* (*t*) and *K* for both test compounds were entered into the ProMISE2 software together with the run mode (head‐to‐tail or tail‐to‐head mode), the *K* value definition (*K_U/L_* or *K_L/U_* value), *V_c_*, $\dot{v}$, and the rotor speed *ω*. Subsequently, the efficiency factor *f'* was determined by curve‐fitting according to Rüttler et al. [21].

##### 2.3.3.3 Calibration Head‐to‐Tail Mode

The results of the four triple determinations for *S_f_* (*t*) and factor *f'* for both test compounds were averaged, and the mean values of all four determinations for both test compounds were plotted in Excel as a function of factor *f'* (*S_f_* (*t*))—with *S_f_ *(*t*) (x‐axis) and factor *f'* (y‐axis). As shown in Section 2.3.2, a linear regression analysis was carried out.

##### 2.3.3.4 Calibration Tail‐to‐Head Mode

The results of the two triple determinations were treated in the same way as in the head‐to‐tail mode and used as calibration for further experiments (Section 2.3.4).

##### 2.3.3.5 Calculation of Angle‐Averaged Lines

Starting with two initial straight lines, the angle‐averaged line was calculated step by step in Excel: (i) the point of intersection of the two initial straight lines was determined, (ii) the average slope was calculated using the angular inclination of the initial straight lines (slope = tan ((arctan (slope 1) + arctan (slope 2)) / (2)), and (iii) using the calculated slope and the point of intersection, the intercept of the angle‐averaged line was determined.

#### 2.3.4 Prediction of Elution Volumes and Peak Widths in CCC Runs With (Slight) Loss of Stationary Phase

##### 2.3.4.1 CCC Separation

The separation of a mix of both test compounds was repeated once in tail‐to‐head mode with a slightly different approach (Section 2.3.3). In brief, *V_s_ *(0) was determined, the effluent was collected in the time slots of 0–20 min, 20–40 min, and 90–110 min, and the volume of displaced stationary phase (*V*_displaced_) was measured (Section 2.3.3).

##### 2.3.4.2 Data Evaluation

For the prediction of the elution time *t* and *w_b_*, the known *K* values (Section 2.3.3) of the first eluting astaxanthin‐5,6‐epoxide (*K*_*L*/*U*_ = 1.18) and the later eluting lutein (*K_L/U_* = 6.56) were used. The stationary phase bleeding rate a (slope of the decrease in *V_s_* (*t*), in mL/min) was calculated from the average of the three collected data points (DP = *V_displaced_* / 20 min, in mL/min). To predict *t*, the dynamic loss of the stationary phase composed of $\dot{v}$ and *a* was included in a modified elution equation (Equation 5):

(5) $$t=(V_{c}+(K\;-\;1)\;\cdot \;V_{s}\;(0))/(\dot{v}+(K\;-\;1)\;\cdot a)\;$$

Based on the known values for *V_c_*, *K*, *V_s_* (0), ${\dot{v}}_{m}$, and a, t was calculated for lutein (Equation 5). Next, t was substituted into $V_{s}\left(t\right)=V_{s}\;\left(0\right)-a\cdot t$, and *S*_*f*_ (*t*) was subsequently calculated (Equation (2)). From the plots of factor *f'* (*S_f_* (*t*)) in tail‐to‐head mode (Section 2.3.3), the corresponding factor $f^{\prime }$ was selected for the predicted *S_f_* (*t*) and entered into the ProMISE2 software together with the known parameters $V_{c}$, K, $\dot{v}$, and the rotor speed *ω* (Section 2.3.3). The resulting simulated elution profiles, including the predicted peak widths, were superimposed with the experimental CCC‐UV/vis elution profiles in Excel and evaluated qualitatively [21].

### 2.4 Phase Composition Analysis With Proton Nuclear Magnetic Resonance Spectroscopy

The procedure in Section 2.3.2 was repeated in a new experiment, and aliquots of the stationary phases from fractions 0–20 min and 300–320 min in head‐to‐tail and tail‐to‐head mode were analysed by proton nuclear magnetic resonance spectroscopy (^1^H NMR) on a Bruker Avance III‐HD 600 MHz NMR spectrometer equipped with a 5 mm TCI CryoProbe Prodigy (Bruker, Rheinstetten, Germany). Sample preparation and data evaluation were carried out according to Zheng et al. (Section S4) [37].

## 3 Results and Discussion

### 3.1 CCC Isolation and Characterization of the Test Compounds

According to the labelling of the oils used, lutein and astaxanthin were present at 20% and 5%, respectively (Section 2.1). As expected [23], the direct spectrophotometric determination of the *K* value (Section 2.2) confirmed that the one of the very nonpolar lutein was well outside the sweet spot range (0.4 ≤ *K*_*L*/*U*_ ≤ 2.5) [15], i.e., a very small one in head‐to‐tail and a very high one in tail‐to‐head mode (Table 1). By contrast, the compound in the astaxanthin oil showed a *K* value in the sweet spot range, which was unrealistic for astaxanthin (Table 1). This produced strong evidence for the presence of another, more polar carotenoid‐related compound (due to the UV/vis measurement). Although unintentional, it was decided to continue with the isolation and characterisation of this compound, as its use as a test compound made it possible to study two carotenoids covering a very wide *K* value range.

**TABLE 1:** Spectrophotometrically determined partition coefficients (*K* values) of lutein oil (20% lutein) and astaxanthin oil (5% astaxanthin) in the *n*‐hexane/benzotrifluoride/acetonitrile (20:7:13, *v*/*v*/*v*) solvent system, including standard deviation.

Sample	*K_U/L_* value	*K_L/U_* value
Lutein oil	0.17 ± 0.03	5.97 ± 0.87
Astaxanthin oil	0.85 ± 0.01	1.18 ± 0.01

*U/L*: ratio of upper to lower phase, corresponds to head‐to‐tail mode. *L/U*: ratio of lower to upper phase, corresponds to tail‐to‐head mode.

**FIGURE 1:**
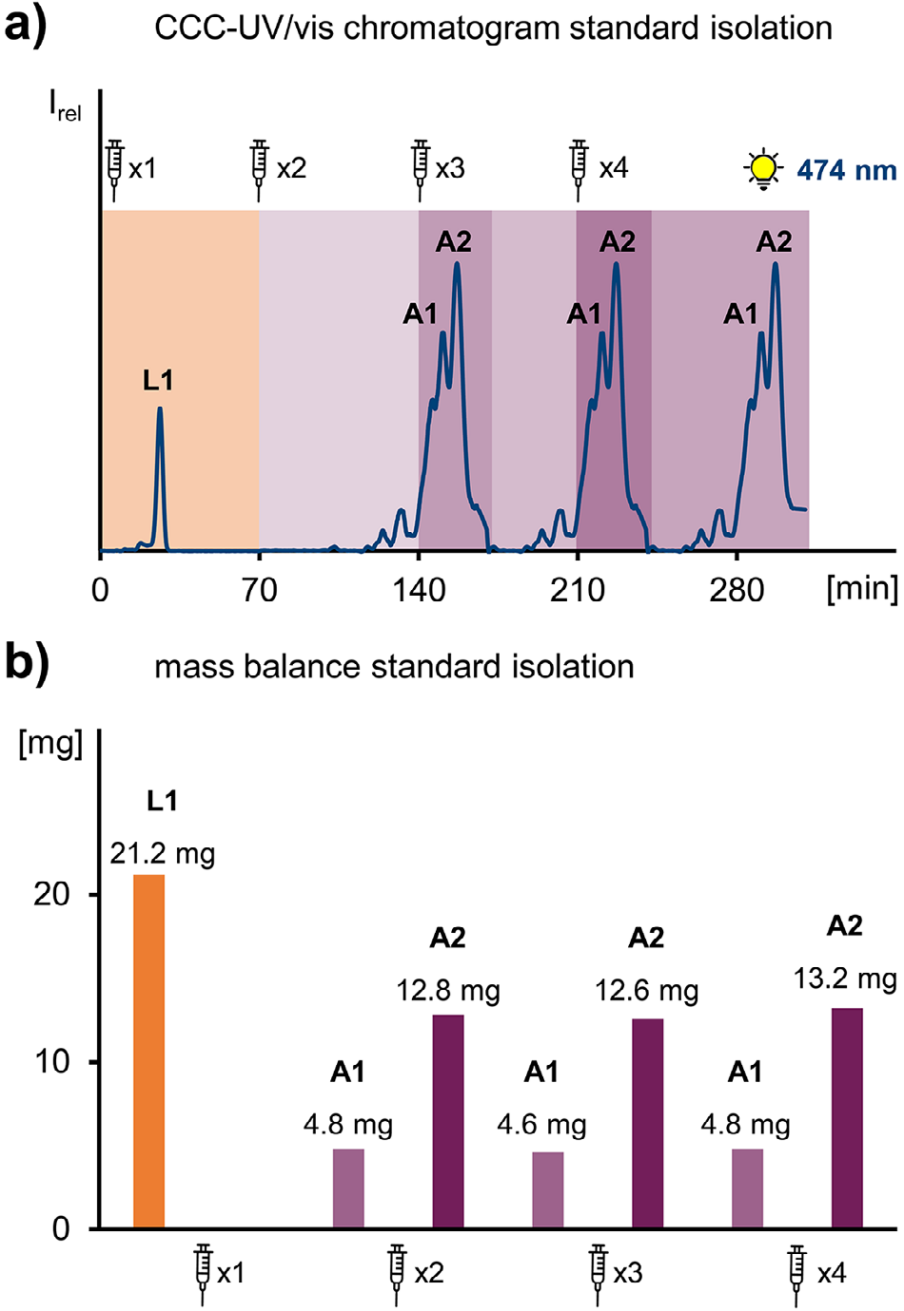
Isolation of test compounds from two safflower oils using countercurrent chromatography (CCC) in co‐current mode: (a) CCC‐UV/vis chromatogram with schematic representation of sample injections every 70 min (four times in sequence) as well as the collected fractions L1 (color: deep orange), A1 (color: deep red‐brown), and A2 (color: red‐brown/amber) and (b) mass balance of collected CCC fractions.

Application of the head‐to‐tail mode in co‐current mode (Section 2.3.1) [32, 33] enabled a complete exchange of the stationary phase after 90 min. However, sequential injections after 70 min were guaranteed not to overlap since the stationary phase was completely replaced with fresh solvent (∼20 min after sample injection) prior to the elution range of the target compounds (∼78–95 min after sample injection, Section 2.3.1). According to this scheme, four subsequent injections were made to enrich the test compounds for further experiments (Figure 1). One single injection of 260 mg lutein oil yielded 21.2 mg lutein (*K*_*U*/*L*_ = 0.17, fraction L1, Figure 1b), whose structure and purity was verified by ultra‐performance liquid chromatography with mass spectrometry (LC‐MS, section S5) (L1: *t* = 2.78 min, C_40_H_56_O_2_, [M+H]^+^ at *m/z* 569.4, [M+H‐H_2_O]^+^ at *m/z* 551.4; purity estimated at ∼92%, Figures S5a and S6a) [38, 39, 40].

By contrast, the astaxanthin oil showed two peaks (corresponding to carotenoids) in the CCC‐UV/vis chromatogram (compounds A1 and A2), which were collected separately (Figure 1a). Specifically, three injections of 260 mg astaxanthin oil each provided ∼4.7 mg A1 (*K*_*U*/*L*_ = 0.67, 14.2 mg in the combined fractions; purity estimated at ∼60%) and ∼12.9 mg A2 (*K*_*U*/*L*_ = 0.89, 38.6 mg in the combined fractions; purity estimated at ∼82%) (Figure 1b). As expected, the LC‐MS data (A1: *t* = 3.84 min and A2: *t* = 4.26 min) did not match an analytical reference standard of astaxanthin (*t* = 2.43 min, [M+H]^+^, *m/z* 597.4, base peak *m/z* 237.0) (Figures S5 and S6) [38, 41, 42]. The LC‐MS spectra of compounds A2 ([M+H]^+^, *m/z* 613.5, Figures S5c and S6c) and A1 ([M+H]^+^, *m/z* 611.4, Figures S5d and S6d) indicated the presence of epoxycarotenoids [43, 44]. According to literature data, isolate A2 was astaxanthin‐5,6‐epoxide (C_40_H_52_O_5_) [45], while isolate A1 was 3',4'‐dehydroastaxanthin‐5,6‐epoxide (C_40_H_50_O_5_) (Figure S6c,d). These structural assignments were supported by fragment ions at *m/z* 445.1 (C_31_H_41_O_2_^+^: elimination of the polyene chain from the epoxidized ring) and *m/z* 371.0 (C_26_H_29_O_2_^+^: cleavage between 10,11‐position and remaining of the charge on the left side of the molecule, Figure S6c), as well as the base peak at *m/z* 237.0 (C_14_H_21_O_3_^+^: cleavage between 10,11‐position and remaining of the charge on the right side of the molecule, confirming the 5,6‐epoxy structure of the analytes, Figure S6c,d) [43, 44, 45]. Since *λ*_max_ (474 nm) corresponded with that of astaxanthin, it was verified that the two 5,6‐epoxides had an intact, conjugated *π*‐system (and a 5,8‐furanoid backbone structure of the analytes could be ruled out) [45]. Furthermore, the additional double bond of 3',4'‐dehydroastaxanthin‐5,6‐epoxide (A1) was confirmed by the presence of *m/z* 237.0 (unchanged epoxy residue) (Figure S6c,d) and its consistent *λ*_max_ (no enlargement of the conjugated *π*‐system) in the non‐functionalized cyclic ring. Subsequent LC‐MS analysis of the (unfractionated) astaxanthin oil sample showed that this sample did not contain any astaxanthin despite the indication of it on the label. Apparently, this vulnerable carotenoid had been oxidatively converted into compounds A1 and A2 during production and/or storage [46, 47]. Accordingly, there is a need for thorough control of active ingredients with carotenoids. However, this unexpected discovery, along with the isolation of astaxanthin‐5,6‐epoxide, allowed us to use two UV/vis active compounds, one of which was inside and one outside the sweet spot range, which was seen as beneficial for later determinations of factor *f*' (Section 3.3).

### 3.2 Investigation of the Gradual Loss of Stationary Phase With the BTF System

**FIGURE 2:**
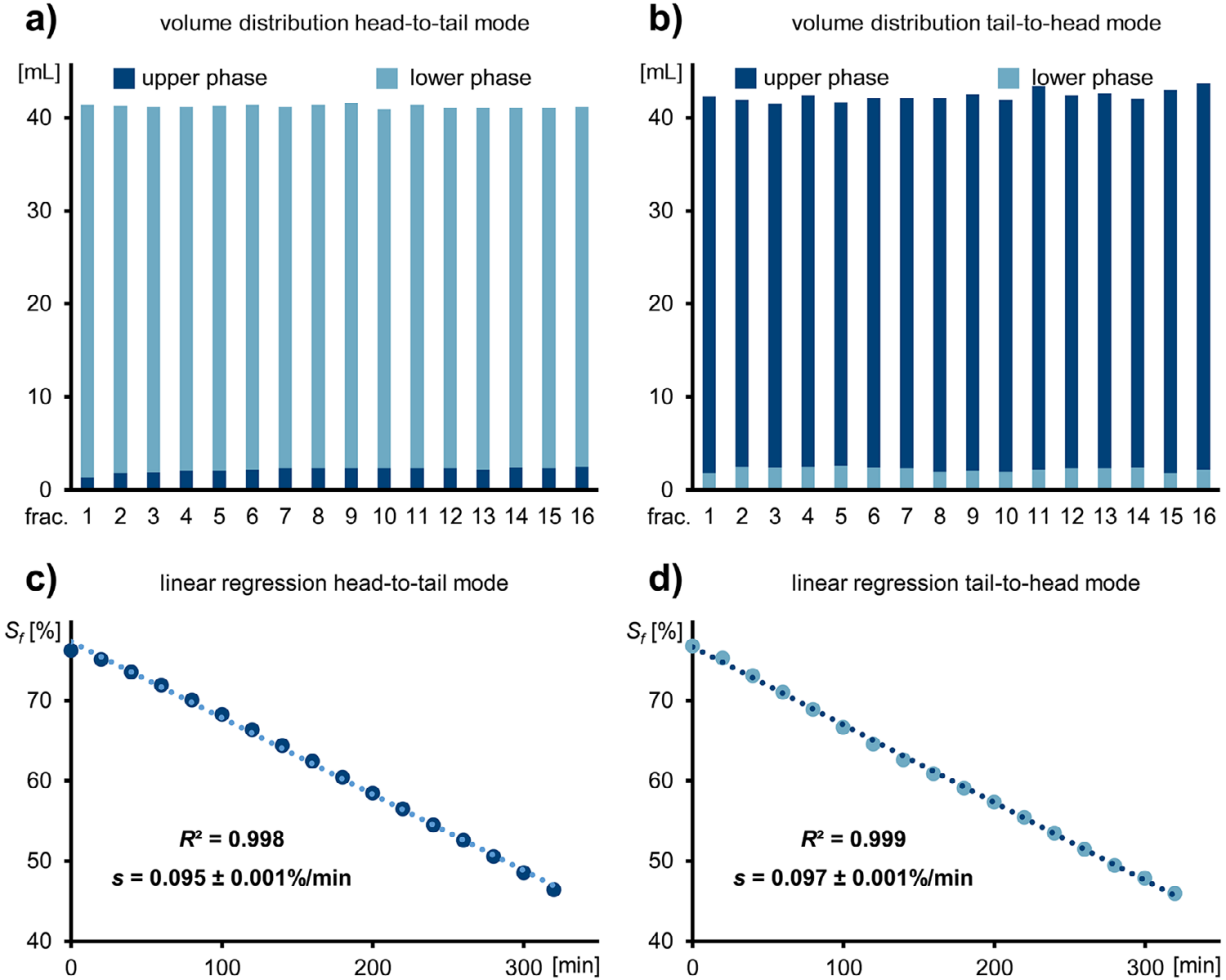
Experiments to determine the gradual loss of the stationary phase in countercurrent chromatography (CCC) using the *n*‐hexane/benzotrifluoride/acetonitrile (20:7:13, *v*/*v*/*v*) solvent system: Volume distribution in the collected CCC fractions (a) in head‐to‐tail mode and (b) in tail‐to‐head mode and linear regression of the loss in the percentile share of stationary phase *s* (c) in head‐to‐tail mode and (d) in tail‐to‐head mode.

Gradual loss of stationary phase was investigated in tail‐to‐head and head‐to‐tail mode without injecting sample (Section 2.3.2). After equilibration, the initial *S_f_* (0) was 76.2% in head‐to‐tail mode and 76.8% in tail‐to‐head mode. Directly after the breakthrough of mobile phase, sixteen 20 min fractions of 40 mL each ($\dot{v}$ *_m_* = 2 mL/min) were taken, and the formation of the second phase was small but directly visible upon closer inspection in each fraction (Figure 2a,b). Volumetric measurements confirmed a permanent slight loss in the percentile share of stationary phase s of 0.095 ± 0.001%/min (*R* ^2^ = 0.998) in head‐to‐tail mode and 0.097 ± 0.001%/min (*R* ^2^ = 0.999) in tail‐to‐head mode (Figure 2c,d, and Tables S1 and S2). In the long term (320 min), this slight loss of stationary phase added up to ∼30% of its initial share, which led to a dramatic drop from *S_f_ *(0) of ∼76.5% to *S*_*f*_ (320) of ∼46.2%. In contrast to a previous study on bleeding with different solvent systems [22], the loss of stationary phase with the BTF system was not exponential but linear in both operating modes. In fact, a linear loss of stationary phase had only been reported once with the solvent system *n*‐heptane/dimethylformamide (1:1, *v*/*v*) applied in tail‐to‐head mode [22]. Apparently, the course of loss of stationary phase is currently unpredictable and needs to be determined individually for biphasic solvent systems.

#### 3.2.1 Observations in Head‐to‐Tail Mode

**FIGURE 3:**
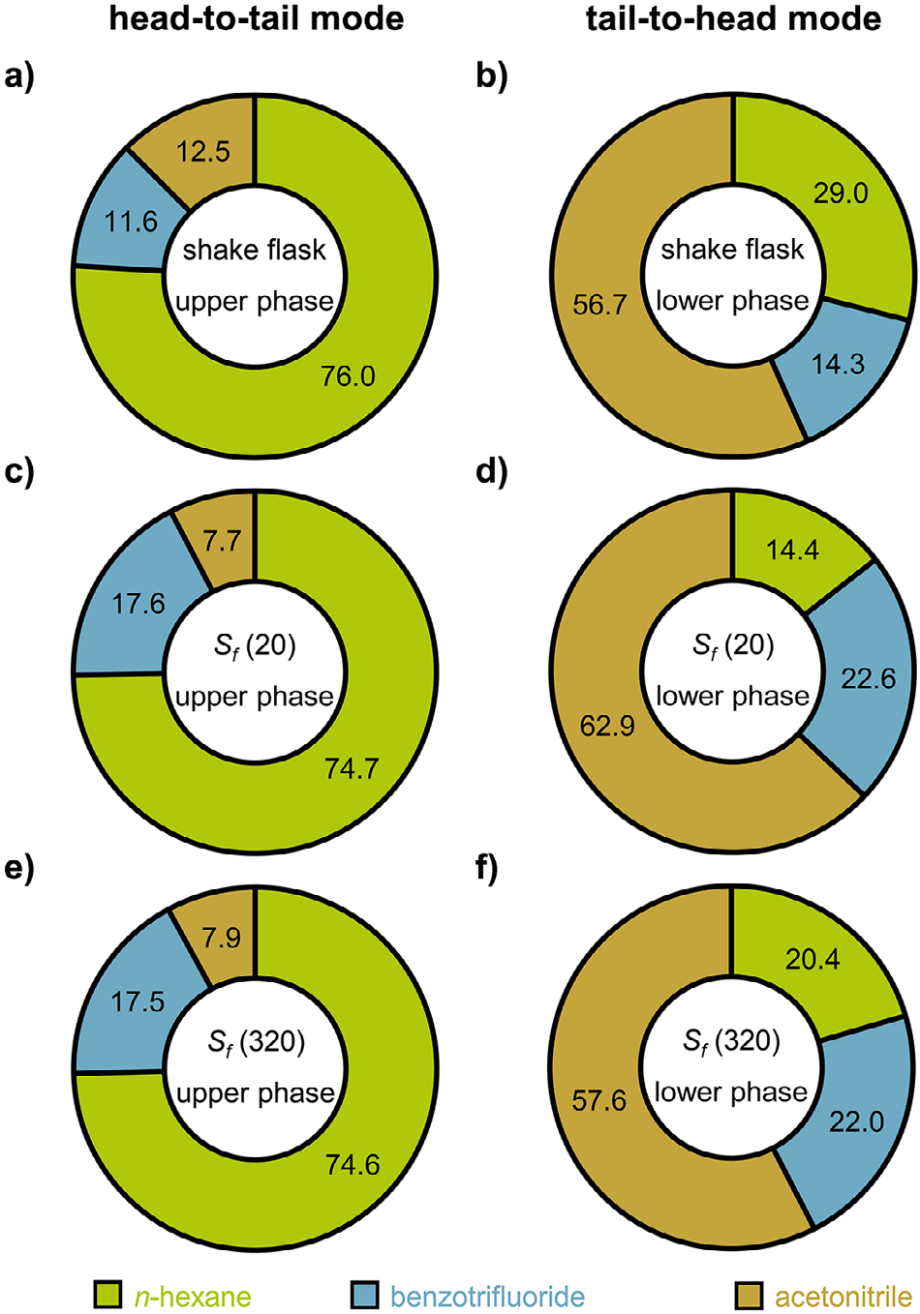
Analytically determined composition of the stationary phase of the *n*‐hexane/benzotrifluoride/acetonitrile (20:7:13, *v*/*v*/*v*) solvent system compared at different stages of a countercurrent chromatographic (CCC) separation in head‐to‐tail (left panel, upper stationary phase) and tail‐to‐head mode (right panel, lower stationary phase): (a, b) Reference values according to Englert et al. [23] from initial shake flask experiments prior to CCC separation, as well as phase composition determined with ^1^H NMR at a time‐dependent loss of the stationary phase *S_f_* (*t*) (c, d) 20 min after equilibration of the solvent system and (e, f) 320 min after equilibration of the solvent system.

Purging after 320 min (and determining *S*_*f*_ (320)) showed that the collected content of the coil became monophasic (Table S1). Obviously, continuous bleeding had changed the composition of the stationary phase of the BTF system over time in a destabilizing way, which may have resulted in a new composition that crossed the binodal line in the ternary phase diagram (Figure S7). To verify this, the procedure was repeated, and samples of the stationary phase were collected at *t* = 0–20 min and *t* = 300–320 min, dissolved in deuterated chloroform, and analysed by ^1^H NMR (Section 2.4) [37]. Remarkably, in fraction *t* = 0–20 min, the composition of the stationary upper phase of 74.7% *n*‐hexane, 17.6% BTF, and 7.7% ACN already differed from the previously known composition of the upper phase (determined directly after the preparation of the biphasic solvent system) of 76.0% *n*‐hexane, 11.6% BTF, and 12.5% ACN (Figure 3a,c) [23]. The stationary and mobile phases of the BTF system had already reached a new equilibrium within the CCC centrifuge under planetary rotation (but were still within the binodal area, Figure S7). Over the course of the separation up to fraction *t* = 300–320 min, the composition of the stationary lower phase remained considerably stable, with 74.6% *n*‐hexane, 17.5% BTF, and 7.9% ACN (Figure 3e). Based on (i) *V_s_ *(0) = 86.7 mL determined in this run, (ii) the stationary phase bleeding rate *a* = 0.05 mL/min (Section 2.3.4), and (iii) the composition of the stationary phase at the final data point, it was possible to derive an overall loss of 18.6% of *n*‐hexane (11.9 mL), 19.0% of BTF (2.9 mL), and 15.4% of ACN (1.0 mL) during the separation. However, the calculation of the exact composition of the nominally monophasic system of *n*‐hexane/BTF/ACN (56.5:16.2:27.3, *v*/*v*/*v*) did not provide any clear indication that it moved outside the binodal region (Figure S7). One possible explanation was that, at the resulting composition of the solvent system, the decrease in *n*‐hexane reduced the density difference between the two liquid phases, resulting in the well‐known phenomenon of a mixture being monophasic under normal conditions, yet becoming biphasic under centrifugal conditions [48].

#### 3.2.2 Observations in Tail‐to‐Head Mode

After 320 mL elution, the purged solvent system remained biphasic and stable, but the share of the stationary phase (*S_f_* (320) = 36.1%) was ∼9.8% (i.e., 11.4 mL stationary phase) lower than at the last experimentally determined data point for the effluent after 320 min (Table S2). This discrepancy was likely caused by changes in the composition of the stationary phase during equilibration. Specifically, already in fraction *t* = 0–20 min, the composition of the (lower) stationary phase determined by ^1^H NMR (14.4% *n*‐hexane, 22.6% BTF, and 62.9% ACN) in the coil deviated from the directly determined composition of 29.0% *n*‐hexane, 14.3% BTF, and 56.7% ACN in a shake flask experiment (Figure 3b,d) [23]. Surprisingly, in fraction *t* = 300–320 min, the composition of the (upper) stationary phase had changed considerably, comprising 20.4% *n*‐hexane, 22.0% BTF, and 57.6% ACN (Figure 3f). Thus, 0% of *n*‐hexane (0 mL), 31.0% of BTF (5.6 mL), and 35.1% of ACN (17.7 mL) were lost during the separation, and the coil contained the overall composition of *n*‐hexane/BTF/ACN (48.7:16.7:34.5, *v*/*v*/*v*) (Figure S7). However, shake flask experiments with the adjusted compositions in fraction *t* = 300–320 min, both head‐to‐tail and tail‐to‐head mode (Section 2.2), resulted only in slight shifts in the *K* values of astaxanthin‐5,6‐epoxide compared to the original BTF systems (Table S3).

### 3.3 Determination of the Efficiency Factor *f*' With the BTF System for a Non‐Constant *S_f_* Value

**FIGURE 4:**
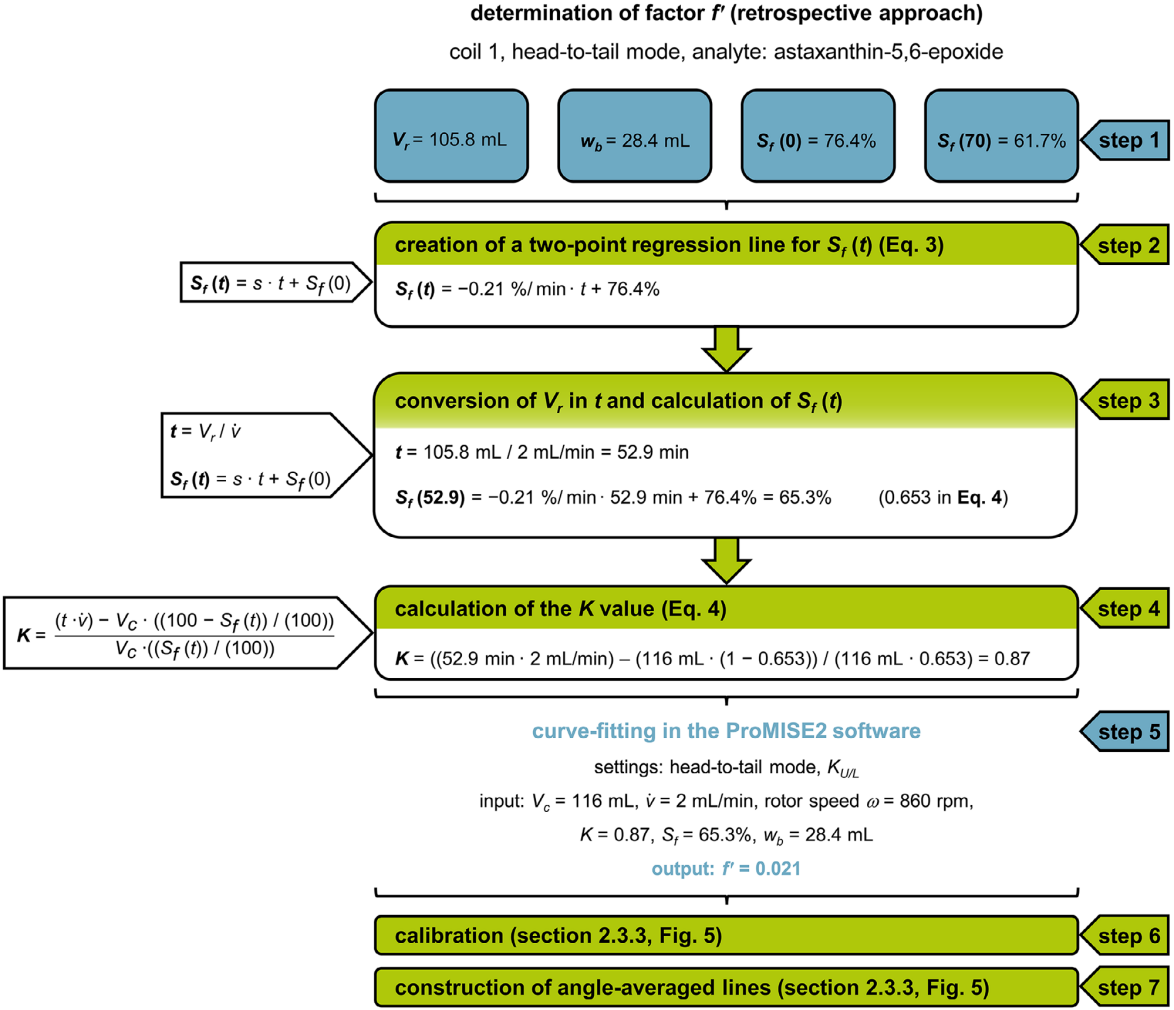
Schematic overview of data evaluation for correcting the *S_f_* value and the *K* value and determining the efficiency factor *f'* using the ProMISE2 software. The general procedure is divided into experimental and software‐related steps (blue) as well as calculation steps (green), with the variable labels given in Section 2.3.3.

The observation of a linear decrease of *S_f_* (*t*) (Section 3.2) made it necessary to consider its impact on factor *f*' in the calculations of the elution volume and peak widths with ProMISE2. Therefore, *S_f_* (*t*) was regressed, and the *K* values of lutein and astaxanthin‐5,6‐epoxide were calculated in five steps based on the currently observed conditions in the coil to eliminate errors caused by bleeding. For example, experimental data indicated an elution volume of 105.8 mL (*t* = 52.9 min) and a peak width at base of *w_b_* = 28.4 mL (step 1, Figure 4). With the experimentally determined *S_f_* (0) of 76.8% and the (by the method duration defined) measured *S*_*f*_ (70) of 61.7% (step 1, Figure 4), the decrease in the percentile share of stationary phase was *s* = ‐0.21%/min (Equation (3)) (step 2, Figure 4). Accordingly, *S_f_* (52.9) was calculated to be 65.3% at this point (step 3, Figure 4). This again allowed us to calculate that the *K* value at 52.9 min corresponded to *K* = 0.87 (step 4, Figure 4). Finally, the relevant variables were inserted into the ProMISE2 software, and curve‐fitting resulted in a perfect overlay between the measured value and the predicted value for *f*' = 0.021 (step 5, Figure 4).

#### 3.3.1 Head‐to‐Tail Mode

**FIGURE 5:**
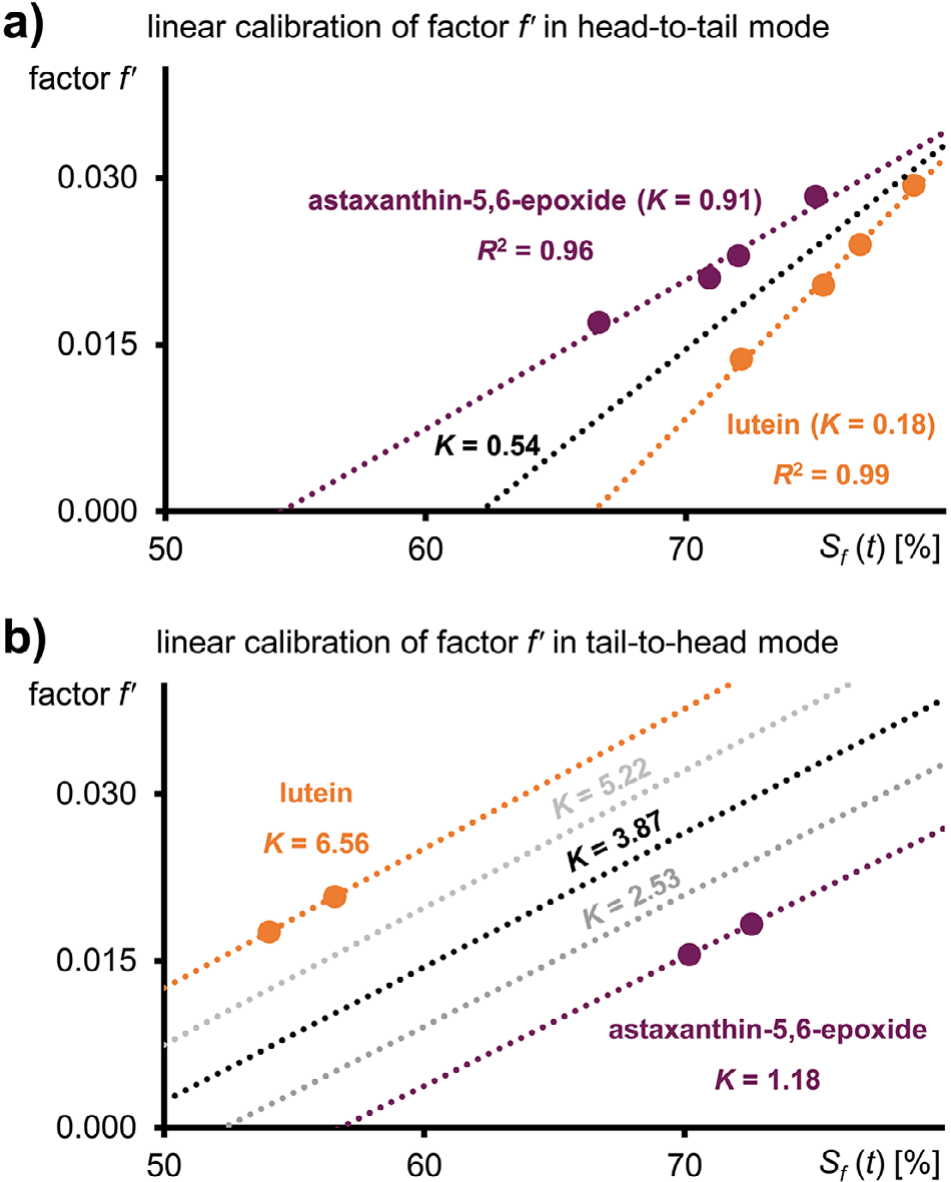
Linear calibration of factor *f'* dependent on *S_f_* (*t*) with lutein and astaxanthin‐5,6‐epoxide, as well as the calculated angle‐averaged lines (examples, each representing a *K* value between the two neighboring outer calibration lines) (a) in head‐to‐tail mode and (b) in tail‐to‐head mode. The calculation of the plots of factor *f'* (*S_f_* (*t*)) and the angle‐averaged lines is described in detail in Section 2.3.3.

Using this approach, factor *f*' was determined in triplicate for all four coils (12 CCC runs) of the multi‐channel CCC device with lutein and astaxanthin‐5,6‐epoxide (Table S4). All coils (on average) yielded different values for *S_f_* (0), but the *S_f_* (*t*) values were all placed on a straight line with good coefficients of determination (lutein: *R* ^2^ = 0.99, astaxanthin‐5,6‐epoxide: *R* ^2^ = 0.96) (step 6, Figures 4 and 5a), which confirmed that the loss of stationary phase was linear. Accordingly, it was derived that the corresponding values of factor *f*' depended on *S_f_* (*t*) and were subsequently described as a function of factor *f*' (*S_f_* (*t*)).

However, the slopes for both compounds were different (Figure 5a), making it reasonable to plot factors *f*' (*S_f_* (*t*)) for each *K* value in order to accurately account for changes in *S_f_* (*t*) in simulations. This was achieved by calculating an angle‐averaged line (step 7, Figure 4). First, the intersection point of the two outer straight lines of lutein (*K* = 0.18) and astaxanthin‐5,6‐epoxide (*K* = 0.91) (i.e., the outer calibration range) was calculated (Section 2.3.3). Secondly, the slopes of the two outer straight lines were converted into their mathematical angles using the arctan function (Section 2.3.3). To calculate the slope of a straight line corresponding to the mean of the *K* values of the two outer analytes ((0.18 + 0.91) / (2) = 0.54), the two angular inclinations were averaged and the slope was calculated with the tan function (slope = tan arctan (slope 1) + arctan (slope 2)) / (2)), Section 2.3.3). Finally, the average slope and the intersection point were substituted into the general equation of a straight line, and a fitting line corresponding to *K* = 0.54 was calculated. The resulting line occurred between the two experimentally determined straight lines of lutein and astaxanthin‐5,6‐epoxide, but it was not the angle bisector (Figure 5a). This is because the angle of the calculated straight line changed according to the tan function, which automatically resulted in unequal intervals between the two initial straight lines on the x‐axis. This approach allowed us to calculate angle‐averaged lines arbitrarily to cover all *K* values within the selected calibration range (see below). Going forward, these graphs are intended to help CCC users determine which factor *f*' needs to be entered in the ProMISE2 software. In this case, the user knows the *K* value of the analyte (a constant in an investigated biphasic solvent system) and the *S*_*f*_ value (determined by linear regression) and simply needs to read the relevant line to determine which factor *f'* to enter in ProMISE2.

#### 3.3.2 Tail‐to‐Head Mode

The procedure was then repeated with two sets of three measurements (six CCC runs) with the same coil (Table S5). Once again, factor *f'* remained on a straight line as a function of *S_f_* (*t*) (Figure 5b). Owing to the higher *K* value of lutein (and the considerably longer elution time of 300 min), the linear calibration theoretically allowed the construction of more angle‐averaged lines within the wider calibration range of *K* = 1.18 and *K* = 6.56 (Figure 5b). In the present example, this was carried out by adding three additional angle‐averaged lines for *K* = 2.53, *K* = 3.87, and *K* = 5.22 (Figure 5b). In summary, we were now able to respond appropriately to fluctuations in *S_f_* (*t*) and to determine a suitable factor *f'* for any separation with the BTF system, which we could then input into the ProMISE2 software.

### 3.4 Prediction of Elution Volumes and Peak Widths of Lutein in the BTF System

Elution times in a theoretical example. To illustrate the impact of *S_f_* (*t*) on the peak position of analytes, three *K* values (0.5, 1.5, and 8.0) were arbitrarily chosen and entered into the modified CCC elution equation (Section 2.3.4). The elution times with (loss in *S_f_* (*t*)) and without bleeding (*S_f_* (0) = const.) were compared under otherwise identical conditions (*V_c_* = 300 mL, *V_s_* (0) = 225 mL (corresponding to *S_f_* (0) = 75%), $\dot{v}$ = 4 mL/min, a stationary phase bleeding rate *a* = 0 or 0.1 mL/min, and 860 rpm). To correctly represent the peak widths *w_b_* in a specific biphasic solvent system for three different *K* values (0.5, 1.5, and 8.0) without (one factor *f'* that applies to all three *K* values) and with bleeding (three different factors *f'* for the selected *K* values), a linear decrease in *f'* (*S_f_* (*t*)) was assumed, with:

- *f'* (*S_f_* (0)) = 0.026 (which corresponds to all three *K* values without bleeding)
- *f'* (*S_f_* (47.5)) = 0.025 (which corresponds to *K* = 0.5 with bleeding)
- *f'* (*S_f_* (101.9)) = 0.024 (which corresponds to *K* = 1.5 with bleeding), and
- *f'* (*S_f_* (398.9)) = 0.017 (which corresponds to *K* = 8.0 with bleeding).

Bleeding slowed down the elution of the analyte with *K* = 0.5 by 0.6 min (47.5 min instead of 46.9 min w/o bleeding, *ΔS_f_* (*t*) = 1.6%) (Figure S8a). For the analyte with *K* = 1.5, bleeding decreased the elution time by 1.2 min to 101.9 min (103.1 min w/o bleeding, *ΔS_f_* (*t*) = 3.4%) (Figure S8b). Presumably, such slight changes would be rather attributed to inexact determinations of experimental parameters (*K* values, *S_f_* values). However, for the analyte with *K* = 8.0, the difference between the elution volume without bleeding (468.8 min) and with bleeding (398.9 min) was 69.9 min (*ΔS_f_* (*t*) = 13.3%) (Figure S8c). Based on these observations, the following conclusion was derived: (i) analytes with *K* = 1 always eluted with one coil volume, (ii) for *K* < 1 the elution was delayed because the analyte interacted almost exclusively with the mobile phase (and its volume increased over time), and (iii) analytes with *K* >> 1 eluted earlier because there was less stationary phase available for mixing and demixing.

#### 3.4.1 Peak Width and Separation Power

In the example shown above, the resolution between the analytes with *K* = 1.5 and 8.0 was affected by the decrease in *S_f_* (*t*) (Figure S8d). Although the distance between both compounds decreased due to the loss in *S_f_* (*t*), this was slightly compensated by the decrease in the efficiency factor *f'* (see plots of *f'* (*S_f_* (*t*))). Thus, the resolution between the two analytes was *R* = 4.83 without bleeding and *R* = 4.58 with bleeding (Figure S8d). This demonstrated that bleeding not only shifted the peak position, but also marginally reduced the separation power [49].

#### 3.4.2 Application to CCC Separations

**FIGURE 6:**
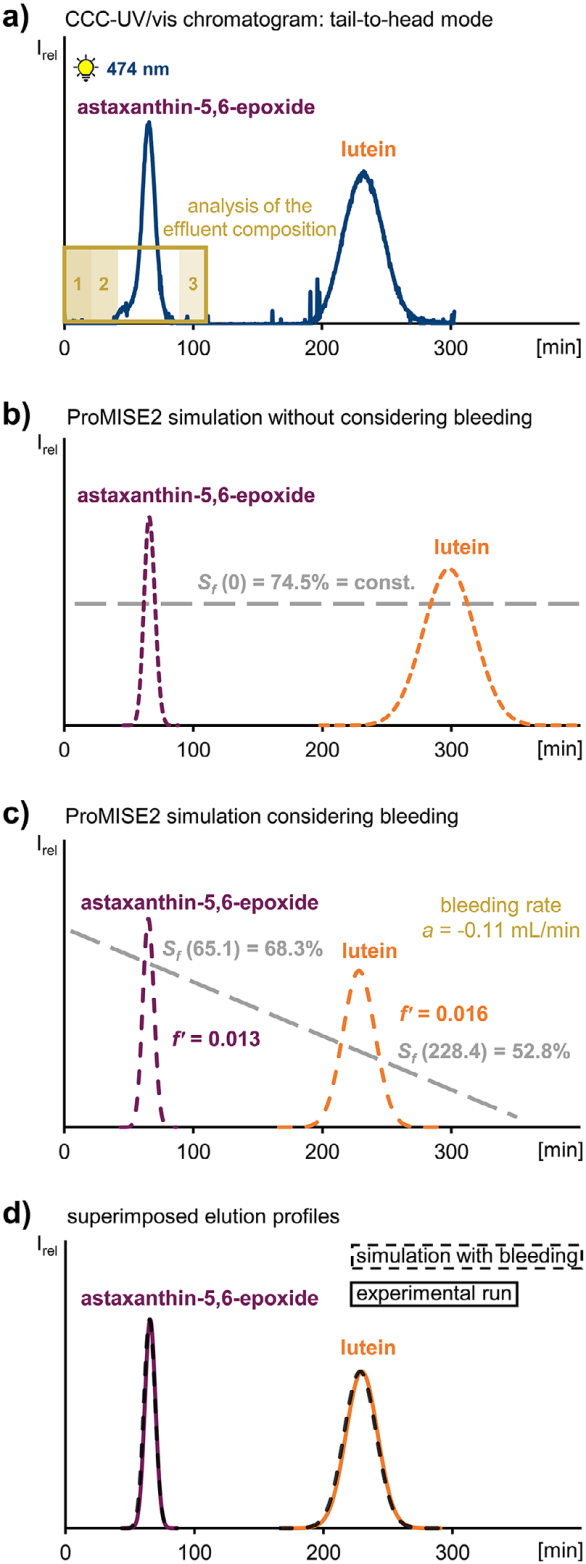
Countercurrent chromatographic (CCC) separation of astaxanthin‐5,6‐epoxide and lutein in tail‐to‐head mode: (a) Experimental CCC run with three collected fractions to determine the stationary phase bleeding rate (*a*) as well as CCC chromatograms simulated using the ProMISE2 software (b) assuming constant retention of the stationary phase (*S_f_* (0) = const., no bleeding), (c) prediction of *S_f_* (*t*) based on *a*, and (d) overlay of the predicted and the experimentally determined chromatogram.

To translate these conclusions into a practical application, a novel approach was developed. Knowledge of the loss in *S_f_* (*t*) made it possible to predict the elution time *t* and peak width *w_b_* of any compound with a known *K* value during a (long) CCC run (Section 2.3.4). In the present example, *S_f_* (0) was determined experimentally, and three arbitrary 20 min fractions were collected (step 1, Figure 6a and Figure S9). Next, the stationary phase bleeding rate of *a* = 0.11 mL/min was calculated from the three arbitrarily selected 20 min fractions (step 2, Figure S9). Subsequently, the elution time *t* of 228.4 min was predicted with the proposed modified CCC elution equation (step 3, Figure S9). In this example, *t* = 228.4 min differed only slightly from the actual *t* of 229.7 min measured throughout the separation. Using *t* = 228.4 min, *S_f_* (228.4) = 52.8% was calculated via *V_s_* (228.4) at the maximum of lutein (step 4, Figure S9). Applying *S_f_* (228.4) = 52.8% in the calibration equation for factor *f'* (Section 3.3) resulted in factor *f'* (*S_f_* (228.4)) = 0.016 for lutein (step 5, Figure S9). Entering *K* = 6.56, factor *f'* = 0.016, and *S_f_* = 52.8%, as well as *V_c_* = 116 mL, $\dot{v}$ = 2 mL/min, and the rotor speed of *ω  *=  860 rpm, ProMISE2 predicted a peak width of *w_b_* = 124.1 mL, which differed only slightly from the experimentally determined peak width of *w_b_* = 121.9 mL (step 6, Figure S9). Visual inspection of the simulated CCC‐UV/vis chromatograms without bleeding (Figure 6b) and with bleeding (Figure 6c) revealed a huge discrepancy in *t* and *w_b_* of lutein. However, the CCC elution profile simulated with bleeding agreed excellently with the experimental data (Figure 6d).

## 4 Conclusions

Thorough investigation of bleeding in CCC exemplified with the BTF system revealed a linear decrease in *S_f_* (*t*), both in head‐to‐tail and tail‐to‐head mode. Linear regression of bleeding allowed a retrospective correction of the *S_f_* value and *K* values. This made it possible to determine the efficiency factor *f'* depending on *S_f_* (*t*). Bleeding was found to have a particularly strong impact on the elution volume and peak width of analytes with comparatively high elution volumes. Hence, a modified CCC elution equation (Equation 5) was introduced, which enabled the prediction of CCC separations with a few simple experimental steps and the software ProMISE2. In practice, the necessary corrections can be made during a (long) CCC run, when a given volume is collected at the start of the separation and used to determine the bleeding rate *a*.

Unlike previously known data for centrifugal partition chromatography (CPC) [30] and CCC [22] with other solvent systems (e.g., *n*‐heptane/ethyl acetate/methanol/water (10:40:10:40, *v*/*v*/*v*/*v*) and 1‐butanol/water (1:1, *v*/*v*)), the decrease in *S_f_* (*t*) of the BTF system was not exponential. However, in the case of an exponential loss of stationary phase, this linear regression model is not applicable and will require an exponential regression of *S_f_* (*t*) [22]. In CPC, bleeding was thoroughly investigated in terms of its temporal course, localization, and possible countermeasures [30, 50, 51]. Such data should also be collected for CCC applications. Accordingly, changes in the stationary phase composition as a result of bleeding in the hydrophobic BTF system had a notable effect, which should also be investigated in hydrophilic solvent systems (e.g., the ethyl acetate/butanol/water solvent system family) [9].

## List of Symbols

*a*
: stationary phase bleeding rate
A1: fraction 1 of the astaxanthin oil
A2: fraction 2 of the astaxanthin oil
*β*
: device parameter for design and hydrodynamics in countercurrent chromatography
*c_s/m_*
: concentration in the stationary/mobile phase
factor *f′*
: efficiency factor in the probabilistic model for immiscible separations and extractions
*K*
: partition coefficient
*K_L/U_*
: partition coefficient between lower and upper phase
*K_U/L_*
: partition coefficient between upper and lower phase
L1: fraction 1 of the lutein oil
*λ*_max_
: absorption maximum
*r*
: coil radius
*R*
: distance between holder and central axis
*s*
: decrease in the percentile share of stationary phase
*σ*
: standard deviation
*S_f_* (*t*): time‐dependent function of the percentile share of stationary phase
*t*
: elution time
*V_c_*
: coil volume
$\dot{v}$ *_m_*
: flow rate of the mobile phase
$\dot{v}$ *_s_*
: flow rate of the stationary phase
*V_s_* (*t*): time‐dependent function of the stationary phase volume
*ω*
: rotor speed
*w_b_*
: peak width at base
*X*
: phase distribution

## Author Contributions

**Felix Rüttler**: Writing – original draft, visualization, investigation, data curation, and methodology. **Rosalie Ormos**: writing – review & editing. **Walter Vetter**: writing – review & editing, supervision, resources, and conceptualization.

## Funding

The authors did not receive funding for this research.

## Conflicts of Interest

The authors declare no conflicts of interest.

## Supporting information




**Supporting File**: jssc70526‐sup‐0001‐SuppMat.pdf.

## Data Availability

The data supporting this study's findings are available from the corresponding author, Walter Vetter, upon reasonable request.
