# Supplementary material for: Prediction of Elution Profiles in Countercurrent Chromatography With a Linear Loss of Stationary Phase
Source: J Sep Sci. 2026 Sep 13;49(9):e70526. doi: 10.1002/jssc.70526 (PMC13572847; doi:10.1002/jssc.70526)
Supplement: Supplementary file 1 [file JSSC-49-e70526-s001.pdf]

Supplementary Information for

Prediction of elution profiles in countercurrent chromatography with a linear loss of stationary phase

Felix Rüttler, Rosalie Ormos, and Walter Vetter*

University of Hohenheim, Institute of Food Chemistry, Department of Food Chemistry (170b), Garbenstraße 28, D-70599 Stuttgart, Germany

* Corresponding author

Walter Vetter

Phone: +49 711 459 24016

E-Mail: walter.vetter@uni-hohenheim.de

ORCID

Felix Rüttler: 0000-0002-5274-4306

Rosalie Ormos: 0009-0007-9254-1902

Walter Vetter: 0000-0002-5592-4265

Section S1*. Solvents and chemicals*

Ethanol (EtOH, ≥99.9%, for HPLC), *n*-hexane (≥96.0%, for HPLC), and methanol (MeOH, ≥99.95%, for LC-MS) were purchased from Th. Geyer (Renningen, Germany). Acetonitrile (ACN, ≥99.5%, for analysis) was obtained from AnalytiChem (Duisburg, Germany), benzotrifluoride (BTF, ≥99%, anhydrous) and dichloromethane (DCM, ≥99.8%, for pesticide residue analysis) were from Sigma-Aldrich (Taufkirchen, Germany). Formic acid (≥99%, for LC-MS) was sourced from VWR International (Darmstadt, Germany) and Nitrogen was produced by an NGM 22 generator (cmc Instruments, Eschborn, Germany).

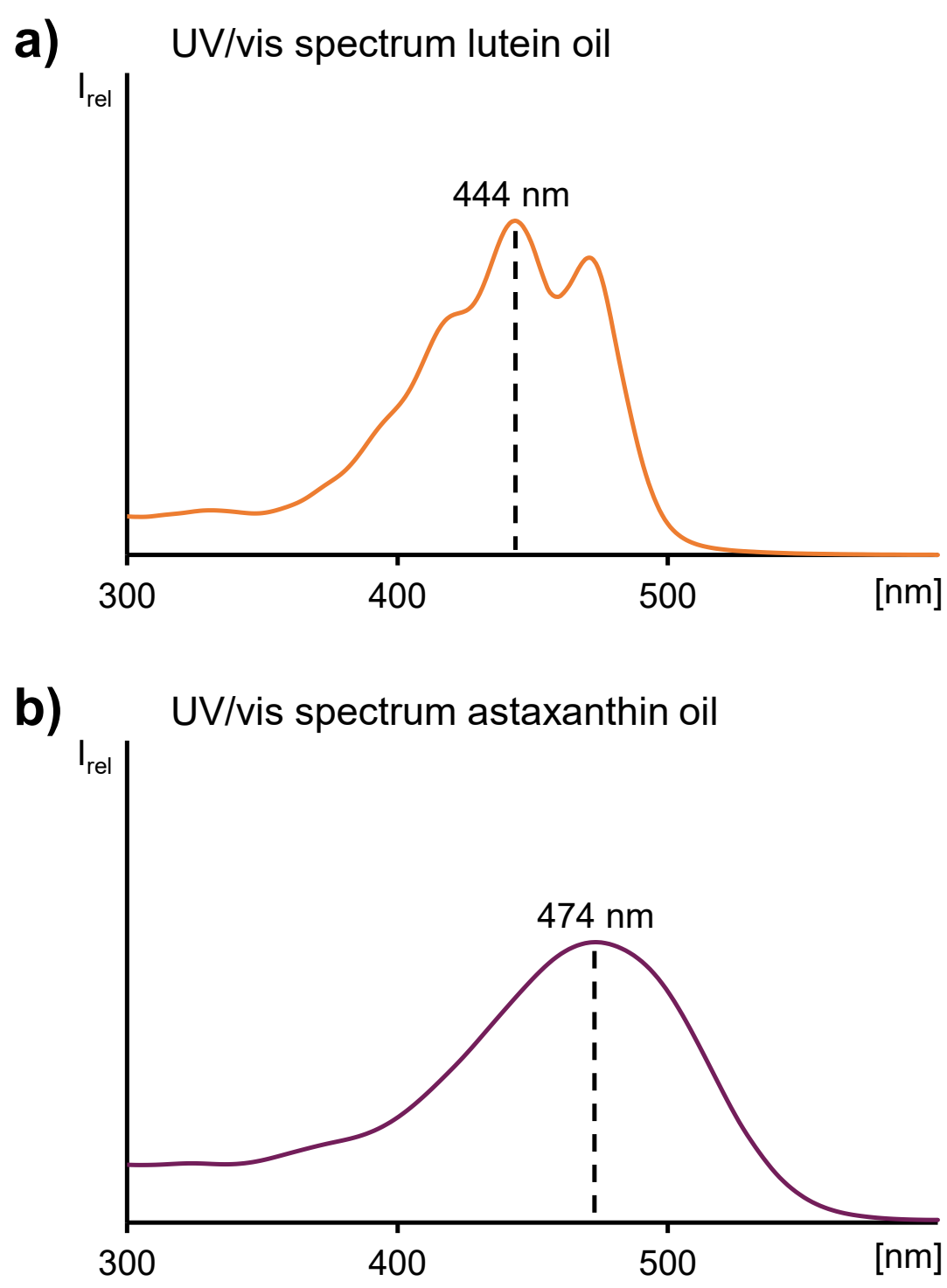

Figure S1. UV/vis spectra of the two safflower oils investigated dissolved in the lower phase of the solvent system *n*-hexane/benzotrifluoride/acetonitrile (20:7:13, *v*/*v*/*v*).

Section S2a. *Spectrophotometric determination of partition coefficients (K values)*

Forty milligrams of both oils were dissolved in 10 mL of ethanol, aliquots of 80 µg were added in triplicate to derivatisation tubes, and the solvent was evaporated under a gentle stream of nitrogen at 38 °C. Subsequently, 1 mL of upper and lower phases of the biphasic solvent system *n*-hexane/BTF/ACN (20:7:13, *v*/*v*/*v*) was added, shaken vigorously, and equilibrated for 10 min. Aliquots of 500 µL were taken from the upper and lower phases, concentrated to dryness under a gentle stream of nitrogen at 38 °C and dissolved in 2 mL of the lower phase of the BTF system. All sample solutions were measured against a lower phase blank with a Genesys 10 UV scanning spectrophotometer (Thermo Scientific, Waltham, MA, USA) in a scanning range from 300 to 600 nm at a medium scanning rate and a wavelength resolution of 3 nm.

Section S2b. *Spectrophotometric determination of K values in variations of the BTF system*

In brief, different compositions of the upper or lower phase (representing the stationary phase) were combined with the standard composition of the opposite phase (representing the mobile phase) [1]. The three selected BTF system variations were: (i) upper phase (regular composition) + lower phase (14.4% *n*-hexane, 22.6% BTF, and 62.9% ACN), (ii) upper phase (regular composition) + lower phase (20.4% *n*-hexane, 22.0% BTF, and 57.6% ACN), and (iii) upper phase (74.6% *n*-hexane, 17.5% BTF, and 7.9% ACN) + lower phase (regular composition).

Section S3. *Countercurrent chromatographic device*

The multi-channel CCC system (**Fig. S2**) was equipped with two dynamically mounted bobbins, which were each fitted with two coils made of multi-wound stainless steel tubes with an internal diameter of 2.1 mm (coil 1: 116 mL, coil 2: 122 mL, coil 3: 114 mL, coil 4: 119 mL) with same β values ($\beta = r/R$, r: coil radius, R: distance between holder and central axis) of β = 0.65 (internal terminal) and β = 0.90 (external terminal) [2]. Coils can be used individually or connected in series in any combination and order. The rotor speed was set to ω = 860 rpm, and the temperature in the CCC centrifuge was kept at ~21 °C using an external cooling device (Blaupunkt, Hildesheim, Germany). Peripheral units (**Fig. S2**) were a ternary beta 50 pump, a flash 10 diode array detector, a Spider C06 mixing unit (all Ecom, Praha, Czech Republic), a 10 mL sample loop (IDEX, Middleboro, MA, USA), and a 203B fraction collector (Gilson, Middleton, WI, USA). CCC chromatograms were simulated with the ProMISE2 software (version 1.2.0.1, Joost de Folter, 2014, joostdefolter.info - ProMISE, last accessed on August 11, 2026) [3].

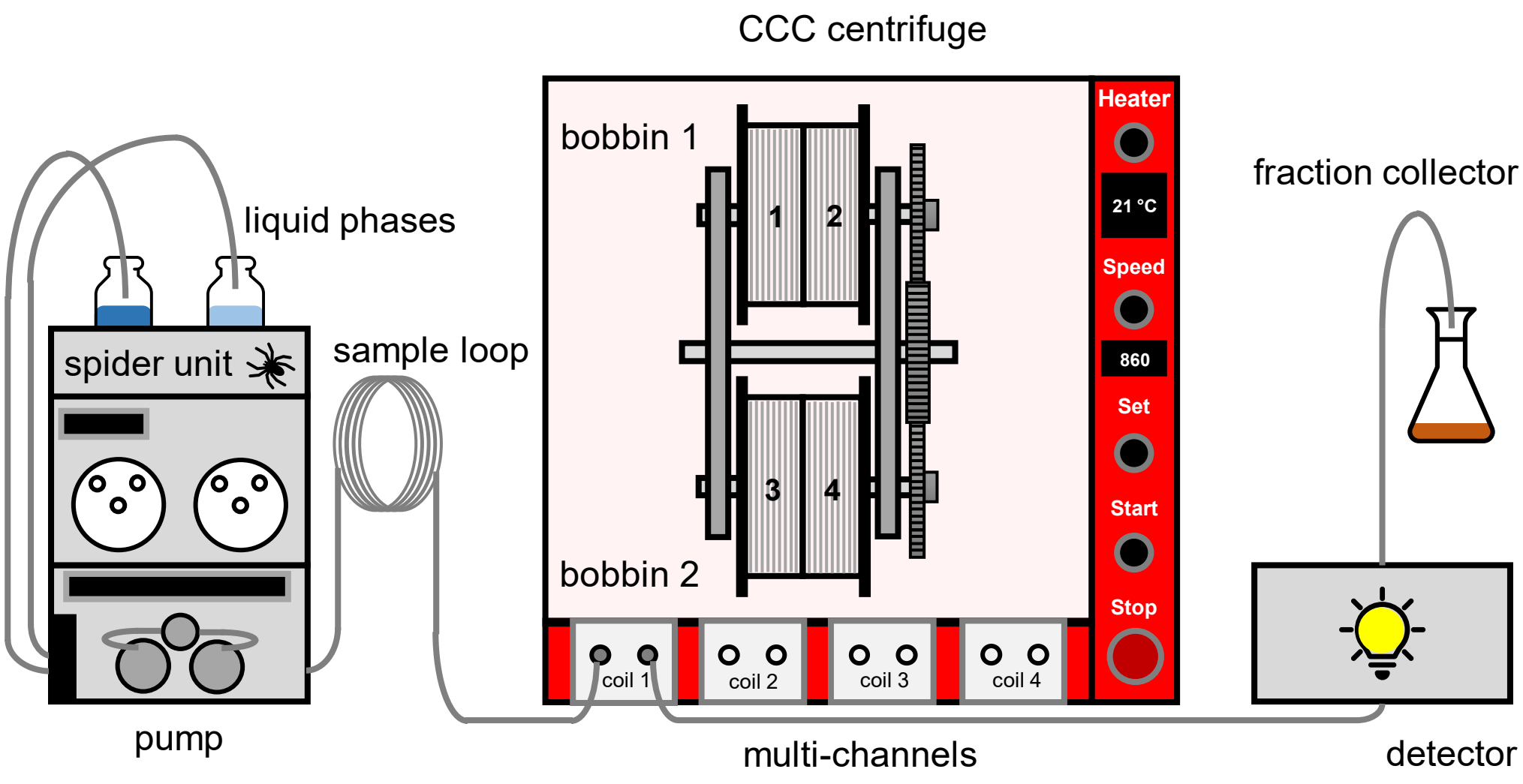

Figure S2. Schematic overview of the experimental countercurrent chromatography (CCC) setup used in this study.

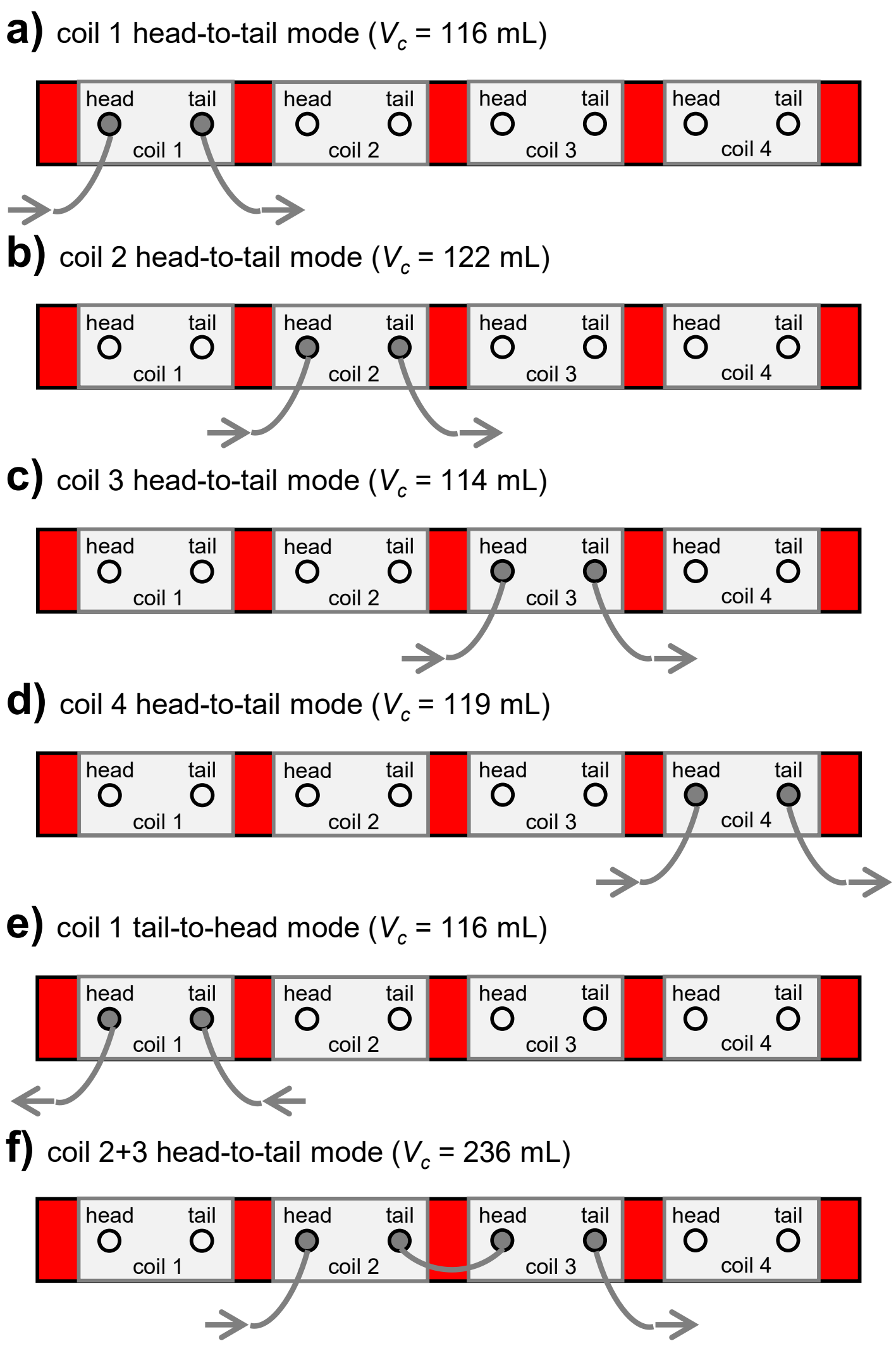

Figure S3. Schematic representation of the multi-channels of the countercurrent chromatography device used in this study, and (**a**-**f**) the respective system configurations used.

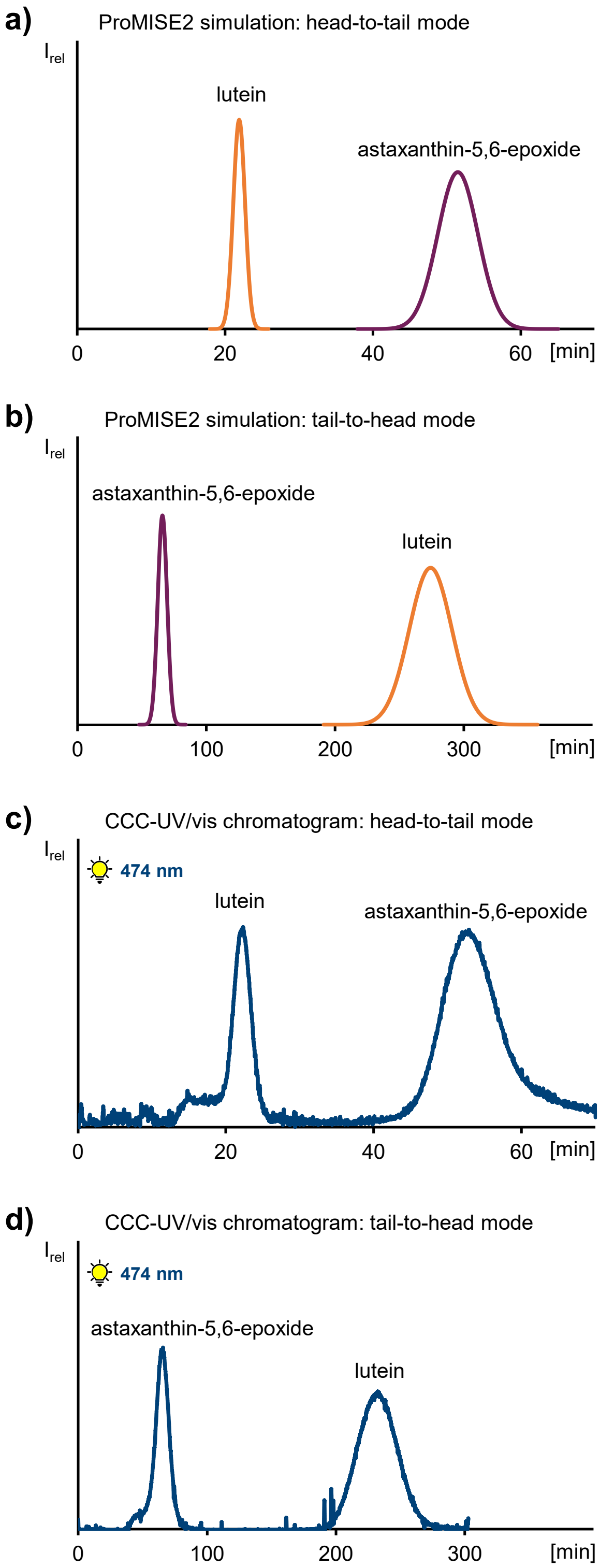

Figure S4. Simulated CCC chromatograms of lutein and astaxanthin-5,6-epoxide (**a**) in head-to-tail mode and (**b**) in tail-to-head mode using ProMISE2 software, as well as experimental CCC-UV/vis chromatograms of both analytes (**c**) in head-to-tail mode and (**d**) in tail-to-head mode.

Section S4. *Phase composition analysis with ^{1}H NMR*

In brief, 50 µL aliquots of the stationary phase were dissolved in 700 µL deuterated chloroform ($CDCl_3$) and determined directly by ^{1}H NMR referenced to the signal of tetramethylsilane (δ scale, 0.0 ppm). The ^{1}H NMR signals of BTF, residual chloroform, ACN, and *n*-hexane were integrated using the SpinWorks 4.2.10.0 software (K. Marat, University of Manitoba, Winnipeg, Canada, 2019). In this context, the singlet of residual chloroform (7.26 ppm, s) did not interfere with the multiplet of BTF (7.45-7.65 ppm, m), the singlet of ACN (1.97 ppm, s), and the protons of *n*-hexane, which were integrated as a sum (4 x CH_2, 8H, 1.23-1.35 ppm, m; 2 x CH_3, 6H, 0.84-0.93 ppm, t). The volume percentages (vol%) of the solvents in the stationary phase were determined according to the method of Amarouche *et al.*, based on the area of the solvent signal (A), the number of protons (n), the molecular weight (MW), and the density (d) of the solvent (**Eq. S1**) [4]:

$$\text{vol\%} = (A / n) \cdot (MW / d) \qquad \text{(S1)}$$

Section S5. *Ultra-performance liquid chromatography with mass spectrometry (LC-MS)*

CCC fractions of the lutein oil, the astaxanthin oil, and the astaxanthin standard were analysed on an Agilent Infinity 1290 II UHPLC system equipped with a G7120A binary pump with solvent degasser, a G7116B column thermostat, and a G7167B autosampler (all Agilent, Waldbronn, Germany) as recently described by Dębczak *et al*. [5]. The UHPLC system was interfaced with a QTrap 6500+ mass spectrometer (Sciex, Darmstadt, Germany) operated in positive atmospheric pressure chemical ionisation (APCI+) mode. Injections (10 µL) were made onto a Gemini NX-C18 column (150 mm length x 2 mm i.d., 3 µm particle size; Phenomenex, Aschaffenburg, Germany), which was maintained at 25 °C. The column was eluted isocratically with MeOH/formic acid (999:1, *v*/*v*), and the flow rate was fixed at 0.2 mL/min (run time: 6 min). The ion source temperature was set to 430 °C, the ion source gas pressure to 30 psi, the curtain gas pressure to 35 psi, the nebuliser current to 2.5 µA, the declustering potential to 65 V, and the entrance potential to 10 V. LC-MS spectra were recorded from 50 to 650 Da (scan rate 200 Da/s) in full scan mode (Q1 scans) and the raw data was analysed with the Analyst software (version 1.7.3, Sciex). The main compounds of both oils were identified by their characteristic mass spectra obtained by in-source fragmentation and their elution time compared to the astaxanthin reference standard (**Figs. S5** and **S6**).

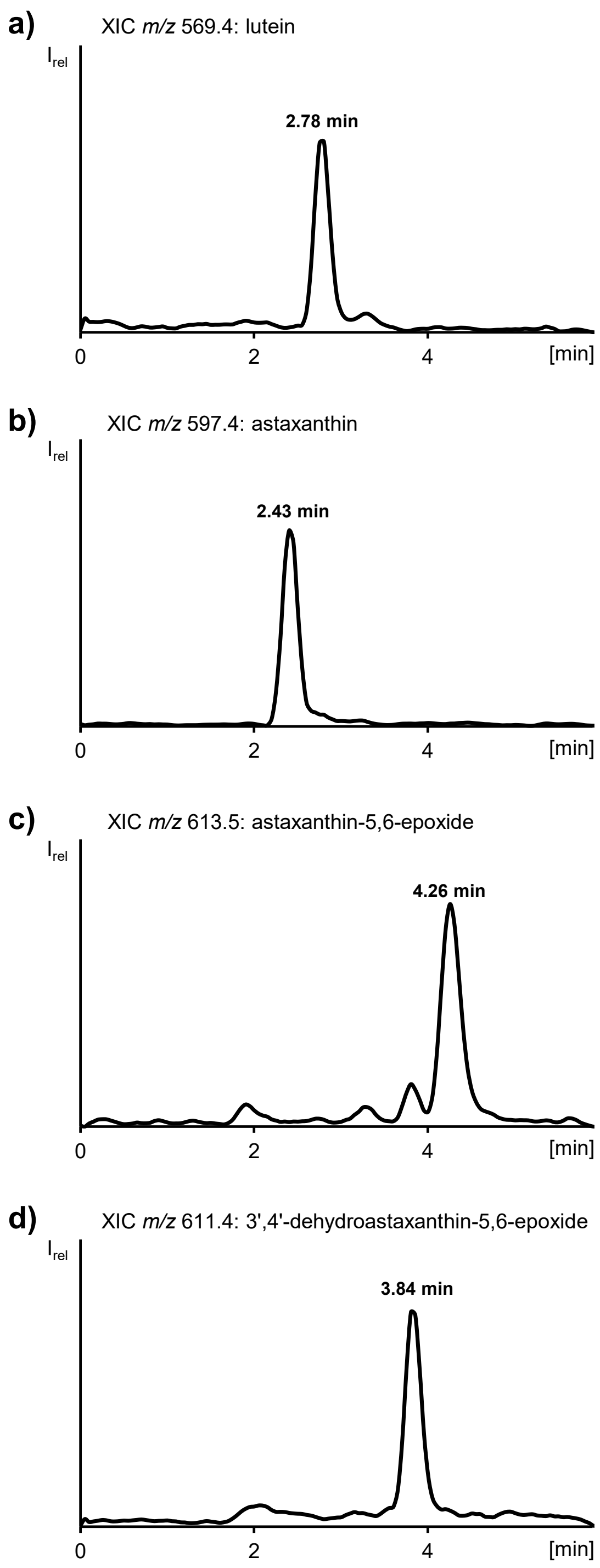

Figure S5. Extracted ion chromatograms (XIC) determined by LC-MS in full scan mode of (**a**) lutein, (**b**) an analytical astaxanthin standard, (**c**) astaxanthin-5,6-epoxide, and (**d**) 3',4'-dehydroastaxanthin-5,6-epoxide.

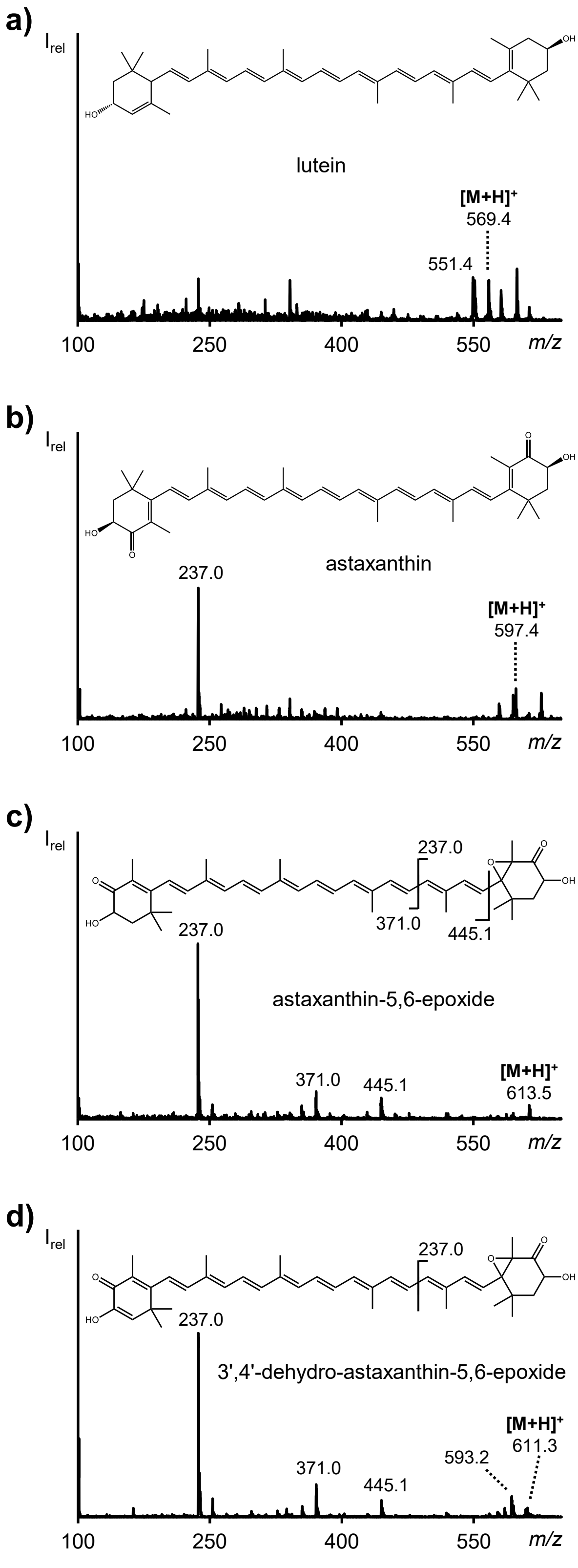

Figure S6. Mass spectra determined by LC-MS in full scan mode of (**a**) lutein, (**b**) an analytical astaxanthin standard, (**c**) astaxanthin-5,6-epoxide, and (**d**) 3',4'-dehydroastaxanthin-5,6-epoxide.

Table S1. Raw data from the experiment to determine the loss of the stationary phase using the *n*-hexane/benzotrifluoride/acetonitrile (20:7:13, *v*/*v*/*v*) solvent system in head-to-tail mode.

t [min]	V_s [mL]	V_m [mL]	V_{s+m} [mL]	V_{cor} [mL]	S_f [%]
0	88.4	27.6	116.0		76.2
20	1.3	40.1	41.4	87.1	75.1
40	1.8	39.5	41.3	85.3	73.5
60	1.9	39.3	41.2	83.4	71.9
80	2.1	39.1	41.2	81.3	70.1
100	2.1	39.2	41.3	79.2	68.3
120	2.2	39.2	41.4	77.0	66.4
140	2.3	38.9	41.2	74.7	64.4
160	2.3	39.1	41.4	72.4	62.4
180	2.3	39.3	41.6	70.1	60.4
200	2.3	38.6	40.9	67.8	58.5
220	2.3	39.1	41.4	65.5	56.5
240	2.3	38.8	41.1	63.2	54.5
260	2.2	38.9	41.1	61.0	52.6
280	2.4	38.7	41.1	58.6	50.5
300	2.3	38.8	41.1	56.3	48.5
320	2.5	38.7	41.2	53.8	46.4
purge	monophasic	monophasic	116.0	n.d.	n.d.

t: time. V_s: volume of the stationary phase. V_m: volume of the mobile phase. V_{s+m}: sum of the mobile and stationary phases. V_{cor}: corrected volume of the stationary phase based on the initially determined V_s with gradual loss over time. S_f: retention of the stationary phase. n.d.: not determined.

Table S2. Raw data from the experiment to determine the loss of the stationary phase using the *n*-hexane/benzotrifluoride/acetonitrile (20:7:13, *v*/*v*/*v*) solvent system in tail-to-head mode.

t [min]	V_s [mL]	V_m [mL]	V_{s+m} [mL]	V_{cor} [mL]	S_f [%]
0	89.1	26.9	116.0		76.8
20	1.8	40.5	42.3	87.3	75.3
40	2.5	39.4	41.9	84.8	73.1
60	2.4	39.1	41.5	82.4	71.0
80	2.5	39.9	42.4	79.9	68.9
100	2.6	39.1	41.7	77.3	66.6
120	2.4	39.7	42.1	74.9	64.6
140	2.3	39.8	42.1	72.6	62.6
160	2.0	40.1	42.1	70.6	60.9
180	2.1	40.4	42.5	68.5	59.1
200	2.0	39.9	41.9	66.5	57.3
220	2.2	41.2	43.4	64.3	55.4
240	2.3	40.1	42.4	62.0	53.5
260	2.3	40.3	42.6	59.7	51.5
280	2.4	39.6	42.0	57.3	49.4
300	1.8	41.2	43.0	55.5	47.8
320	2.2	41.5	43.7	53.3	46.0
purge	41.9	74.1	116.0	41.9	36.1

t: time. V_s: volume of the stationary phase. V_m: volume of the mobile phase. V_{s+m}: sum of the mobile and stationary phases. V_{cor}: corrected volume of the stationary phase based on the initially determined V_s with gradual loss over time. S_f: retention of the stationary phase. n.d.: not determined.

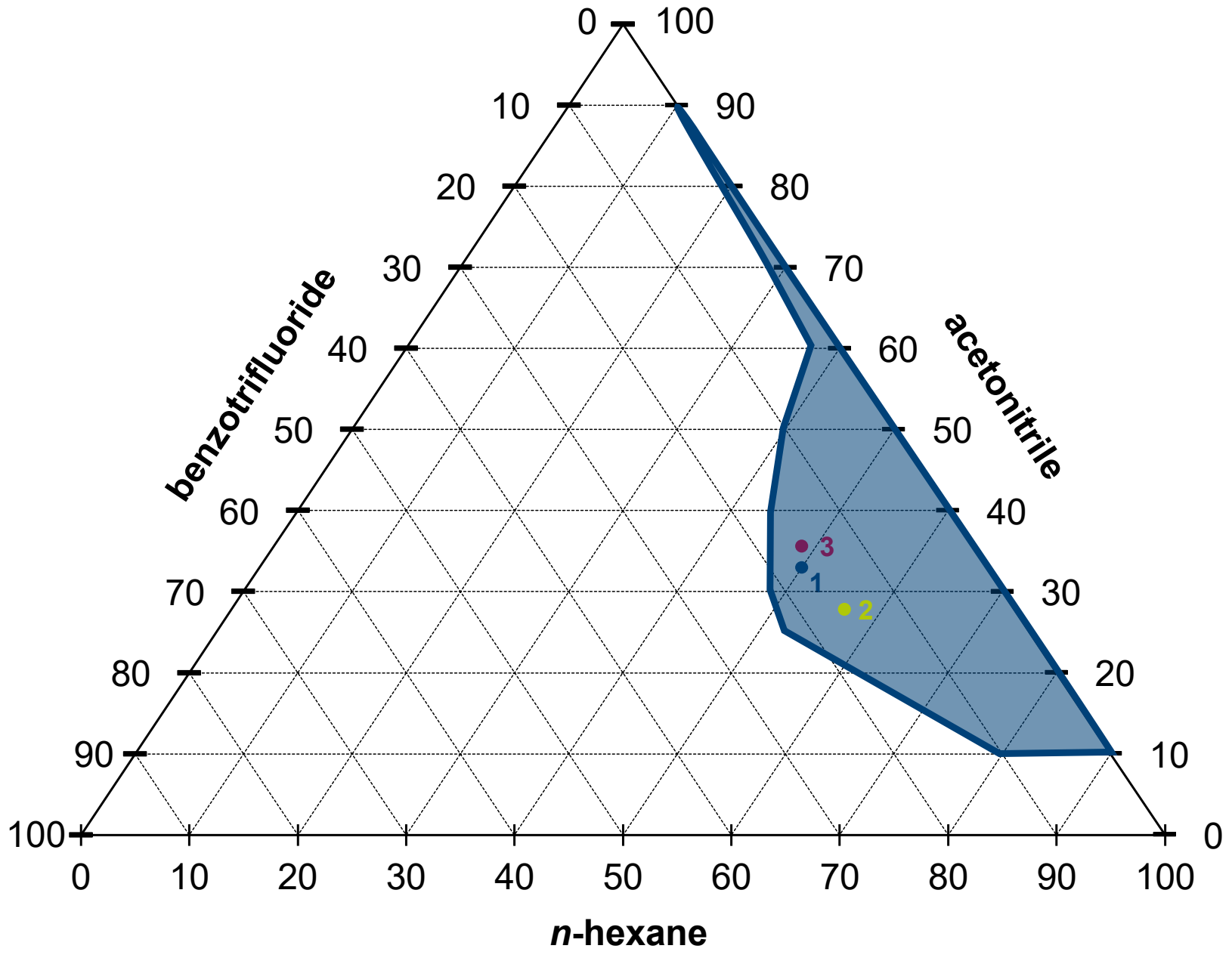

Figure S7. Phase diagram of the ternary mixture of *n*-hexane/benzotrifluoride/acetonitrile, including a binodal line (blue) within which biphasic solvent systems are present [1]. The diagram shows (**1**) the original composition 20:7:13 (*v*/*v*/*v*) and the calculated overall composition of the solvent system after 320 min (**2**) in head-to-tail mode 56.5:16.2:27.3 (*v*/*v*/*v*), and (**3**) tail-to-head mode 48.7:16.7:34.5 (*v*/*v*/*v*).

Table S3. Spectrophotometrically determined partition coefficients (*K* values) of safflower/astaxanthin oil (5% astaxanthin according to label) in variations of the *n*-hexane/benzotrifluoride/acetonitrile (20:7:13, *v*/*v*/*v*) solvent system, including standard deviation.

variation	$K_{U/L}$ value	$K_{L/U}$ value
#1	0.85 ± 0.01	1.18 ± 0.01
#2	0.99 ± 0.00	1.01 ± 0.00
#3	1.02 ± 0.02	0.98 ± 0.02
#4	0.99 ± 0.01	1.01 ± 0.01

#1: both phases - regular composition (20:7:13, *v*/*v*/*v*). #2: upper phase - regular composition + lower phase - 14.4% *n*-hexane, 22.6% benzotrifluoride, and 62.9% acetonitrile. #3: upper phase - regular composition + lower phase - 20.4% *n*-hexane, 22.0% benzotrifluoride, and 57.6% acetonitrile. #4: upper phase - 74.6% *n*-hexane, 17.5% benzotrifluoride, and 7.9% acetonitrile + lower phase - regular composition. *U/L*: ratio of upper to lower phase, corresponds to head-to-tail mode. *L/U*: ratio of lower to upper phase, corresponds to tail-to-head mode.

Table S4. Data related to the chromatographic parameters used to determine factor *f'* in triplicate for four variably controllable coils of a multi-channel countercurrent chromatography device in head-to-tail mode.

#	S_f (0)	S_f (70)	V_r L1	w_b L1	*K* L1	*f'* L1	V_r A2	w_b A2	*K* A2	*f'* A2
1	76.4	61.7	44.4	9.8	0.14	0.012	105.8	28.4	0.87	0.016
	75.8	63.1	44.4	8.9	0.14	0.015	105.1	27.6	0.86	0.017
	76.3	65.6	44.4	9.8	0.15	0.014	105.8	28.4	0.87	0.018
2	79.8	69.8	45.3	9.8	0.18	0.020	111.1	28.4	0.88	0.023
	77.0	68.1	45.3	8.7	0.15	0.019	110.2	29.3	0.86	0.019
	78.4	68.8	46.2	8.9	0.17	0.022	109.3	28.4	0.85	0.021
3	82.0	73.1	42.1	9.8	0.20	0.025	109.5	30.8	0.95	0.024
	78.9	68.0	44.9	9.1	0.20	0.024	108.8	28.1	0.94	0.022
	78.9	68.0	44.2	9.1	0.19	0.023	109.5	28.1	0.94	0.023
4	83.3	77.5	42.1	9.8	0.21	0.029	115.8	31.5	0.97	0.030
	80.6	72.9	44.9	9.1	0.20	0.029	115.8	28.8	0.96	0.028
	79.5	70.8	45.6	8.4	0.20	0.030	115.8	28.1	0.96	0.027

L1: lutein. A2: astaxanthin-5,6-epoxide. S_f (0): retention of the stationary phase at 0 min [%]. S_f (70): retention of the stationary phase at 70 min [%]. V_r: retention volume [mL]. w_b: peak width at base [mL]. *K*: partition coefficient. *f'*: efficiency factor.

Table S5. Data related to the chromatographic parameters used to determine factor *f'* in triplicate (in two different weeks) for a single, variably controllable coil of a multi-channel countercurrent chromatography device in tail-to-head mode.

#	S_f (0)	S_f (300)	V_r A2	w_b A2	*K* A2	*f'* A2	V_r L1	w_b L1	*K* L1	*f'* L1
1	74.5	46.1	131.3	40.6	1.19	0.015	465.6	118.8	6.74	0.018
	77.2	55.1	131.3	43.8	1.18	0.016	459.4	131.3	5.91	0.018
	77.7	42.4	131.3	40.6	1.19	0.016	481.3	121.9	7.38	0.017
2	79.3	52.6	131.3	43.8	1.18	0.019	496.2	124.0	6.73	0.021
	78.4	50.0	131.3	40.6	1.18	0.018	468.6	120.0	6.41	0.020
	78.4	49.1	131.3	40.6	1.18	0.018	454.5	111.4	6.19	0.021

A2: astaxanthin-5,6-epoxide. L1: lutein. S_f (0): retention of the stationary phase at 0 min [%]. S_f (300): retention of the stationary phase at 300 min [%]. V_r: retention volume [mL]. w_b: peak width at base [mL]. *K*: partition coefficient. *f'*: efficiency factor.

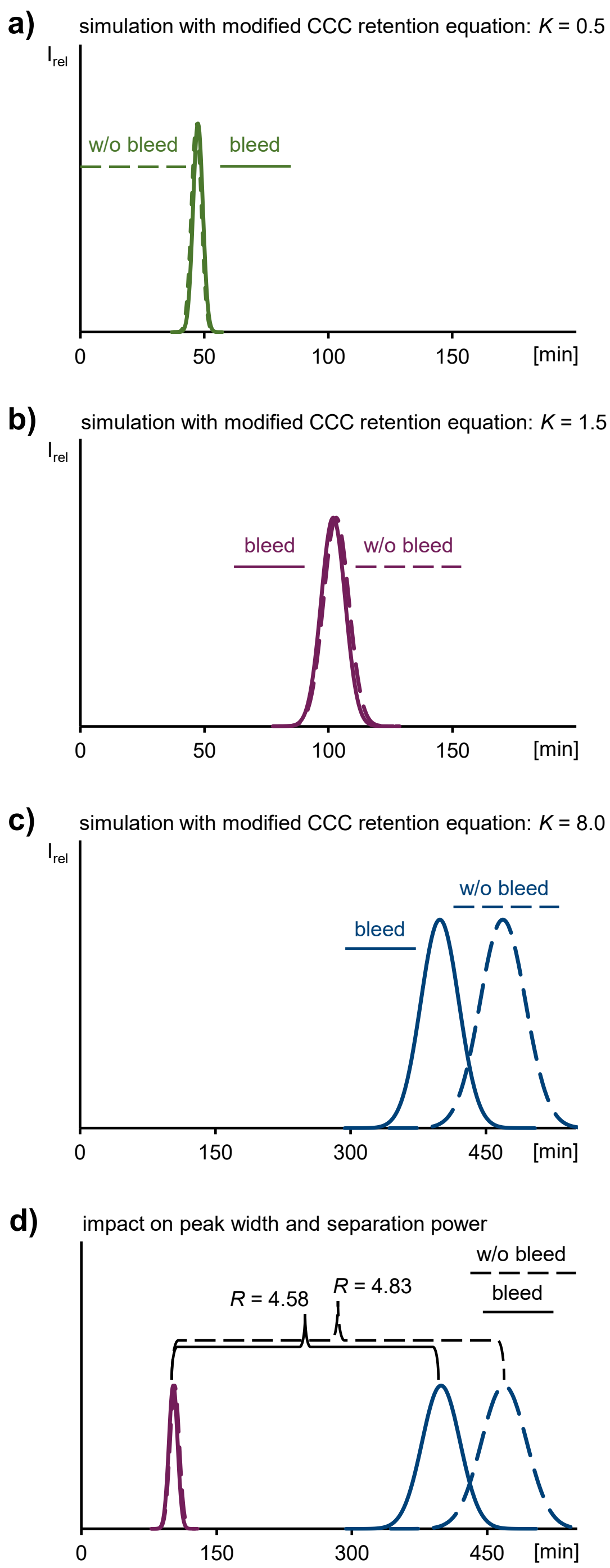

Figure S8. Simulated countercurrent chromatography (CCC) chromatograms in tail-to-head mode using the modified CCC retention equation for biphasic solvent systems both with and without bleeding at different partition coefficients (*K*): (**a**) $K = 0.5$, (**b**) $K = 1.5$, and (**c**) $K = 8.0$ as well as (**d**) a comparison of the resolution between analytes with $K = 1.5$ and 8.0, with and without bleeding.

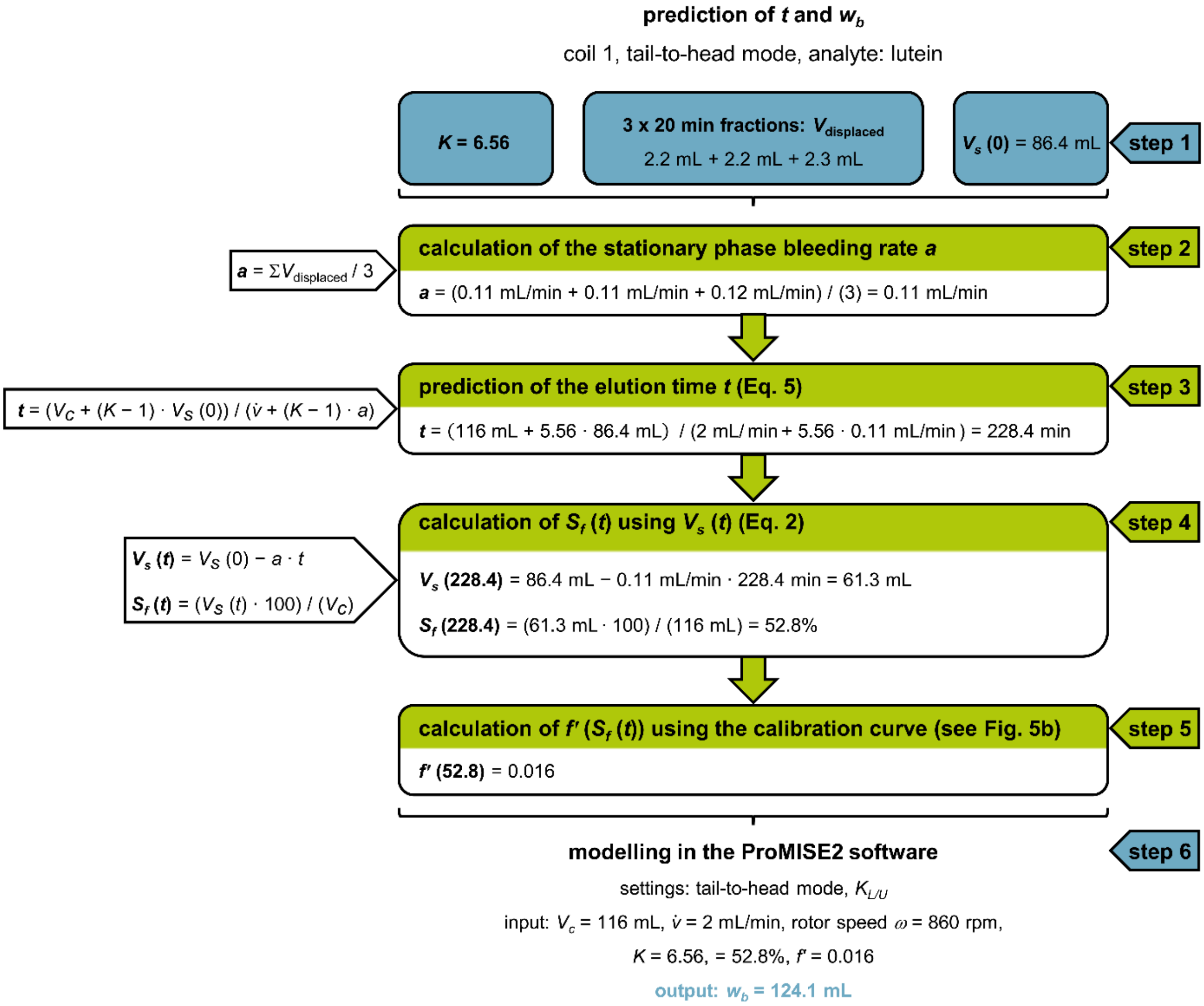

Figure S9. Schematic overview of the approach to predict elution time and peak width at base in countercurrent chromatographic separations with loss of stationary phase using the ProMISE2 software. The general procedure is divided into experimental and software-related steps (blue) as well as calculation steps (green), with the variable labels given in **section 2.3.4**.

References

[1] M. Englert, S. Hammann, W. Vetter, Isolation of β-carotene, α-carotene and lutein from carrots by countercurrent chromatography with the solvent system modifier benzotrifluoride, J. Chromatogr. A 1388 (2015) 119–125. https://doi.org/10.1016/j.chroma.2015.02.020.

[2] M. Englert, L. Brown, W. Vetter, Heart-cut two-dimensional countercurrent chromatography with a single instrument, Anal. Chem. 87 (2015) 10172–10177. https://doi.org/10.1021/acs.analchem.5b02859.

[3] F. Rüttler, W. Vetter, A simple approach to predict device-specific peak widths in countercurrent chromatography, J. Chromatogr. A 1746 (2025) 465803. https://doi.org/10.1016/j.chroma.2025.465803.

[4] N. Amarouche, M. Giraud, L. Forni, A. Butte, F. Edwards, N. Borie, J.-H. Renault, Two novel solvent system compositions for protected synthetic peptide purification by centrifugal partition chromatography, J. Chromatogr. A 1337 (2014) 155–161. https://doi.org/10.1016/j.chroma.2014.02.052.

[5] A. Dębczak, K. Lehnert, V. Bauer, R. Ormos, F. Rüttler, W. Vetter, Isolation and purification of solanesol from post-distillation residue of burley tobacco leaves by countercurrent separation methods, Sep. Purif. Technol. 364 (2025) 132398. https://doi.org/10.1016/j.seppur.2025.132398.
